# Lipid-rich necrotic core of the carotid plaque and the risk of major adverse cardiovascular and cerebrovascular events: a meta-analysis and systematic review

**DOI:** 10.7717/peerj.21214

**Published:** 2026-05-06

**Authors:** Yingqiu Sun, Bingrui Zhang, Leyan Hu, Qinhua Fan, Yuqi Zhang, Wenkai Guo, Qingxiao Li, YaWei Du, Shengxian Wu

**Affiliations:** 1 Beijing University of Chinese Medicine Affiliated Dongzhimen Hospital, Beijing, China; 2 China-Japan Friendship Hospital, Beijing University of Chinese Medicine, Beijing, China

**Keywords:** MACCEs, Lipid-rich necrotic core, Carotid plaque, Stroke

## Abstract

**Background:**

Carotid atherosclerosis drives major adverse cardiovascular and cerebrovascular events (MACCEs), but the role of lipid-rich necrotic core (LRNC) remains controversial. We aimed to clarify this *via* systematic review and meta-analysis.

**Methods:**

PubMed, Embase, Web of Science, and Cochrane were searched for studies reporting LRNC prevalence or size and MACCEs from inception to March 1, 2026. Study quality was assessed using the Agency for Healthcare Research and Quality (AHRQ) and Newcastle-Ottawa Scale (NOS) tools.

**Results:**

Our meta-analysis included 18 studies, involving 5,682 patients and 6,257 carotid plaques. Our results showed that in individuals with lipid-core-containing carotid plaques, the cumulative incidence of MACCEs over a mean follow-up of ∼32.2 months (range 3.1–61.2 months) was approximately 7.6% (95% CI [0.059–0.098]). When a stroke occurred, the probability of it occurring on the same side as a lipid-core-containing plaque was approximately 58.0% (95% CI [0.427–0.720]). Moreover, the presence of a lipid core in carotid plaques was a risk factor for MACCEs, increasing their incidence (HR: 1.570, 95% CI [1.006–2.450]; OR: 2.042, 95% CI [1.474–2.828]). Compared with patients without MACCEs, those with MACCEs had larger mean LRNC volumes (in mm^3^) (MD: 8.284 mm^3^, 95% CI [1.353–15.215]), larger maximum LRNC volumes (in mm^2^) (MD: 8.68 mm^2^, 95% CI [4.095–13.265]), and a higher proportion of LRNC in the plaque (in %) (MD: 0.471%, 95% CI [0.073–0.868]). Additionally, patients with MACCEs had a higher proportion of lipid core volume in the plaque (in %) (MD: 2.283%, 95% CI [0.354–4.211]).

**Conclusions:**

Despite limited studies, LRNC presence and size are MACCE risk factors, suggesting therapeutic targeting prevents MACCEs.

## Introduction

Major adverse cardiovascular and cerebrovascular events (MACCEs), including acute myocardial infarction, sudden cardiac death, and ischemic stroke, are primary causes of reduced life expectancy and diminished quality of life worldwide (Barbarawi et al., 2019; Ge et al., 2024). The Global Burden of Disease, Injuries, and Risk Factors Study (GBD) has identified ischemic heart disease and ischemic stroke as the leading and third leading causes of global mortality, respectively, with the number of disability-adjusted life years (DALYs) and deaths still on the rise (Roth et al., 2018; Roth et al., 2020). In recent years, there has been a growing call from researchers to focus on the disease burden and risk factors associated with cardiovascular and cerebrovascular events (Mensah, Roth & Fuster, 2019; Vaduganathan et al., 2022). Therefore, the early identification, monitoring, and necessary treatment of risk factors to prevent these events are of paramount importance.

Atherosclerosis is a significant driver of MACCEs (Fan & Watanabe, 2022). For instance, carotid atherosclerosis is believed to be the cause of 10% to 15% of ischemic strokes globally (Schermerhorn et al., 2019). Moreover, the rupture of unstable atherosclerotic plaques can lead to thromboembolic events, such as myocardial infarction (MI) (Tsao et al., 2023; Shamsuzzaman et al., 2024). Atherosclerosis is characterized by the accumulation of lipids and fibrous elements in large arteries and is a chronic inflammatory disease (Lusis, 2000). In recent years, the composition of carotid plaques has also been recognized as a predictor of cardiovascular and cerebrovascular events, even surpassing plaque burden in importance (Sun et al., 2017). Specifically, the accumulation of cellular debris and lipids in necrotic cells leads to the formation of lipid cores, which are often accompanied by the infiltration of inflammatory cells in carotid plaques (Xu et al., 2021). The relationship between the lipid core, a significant component of carotid plaques, and MACCEs remains a topic of interest (Tan et al., 2023). For example, the histological identification of vulnerable plaques includes the presence of a large LRNC, which is associated with sudden cardiac death (Porambo & De Marco, 2020). The LRNC is thought to be closely related to plaque vulnerability and can serve as an independent risk factor for plaque rupture and a predictor of stroke (Ota et al., 2009; Xia et al., 2017; Saba et al., 2019; Deng et al., 2020). However, some recent studies have suggested that the size of the LRNC, rather than its mere presence, is predictive of stroke and coronary heart disease (Bos et al., 2021). Therefore, we aim to gain a deeper understanding of the relationship between the lipid core and cardiovascular and cerebrovascular events, including the cumulative incidence of MACCEs in individuals with LRNC and the relationship between the presence and size of the lipid core and these events.

Common imaging methods for assessing LRNC in atherosclerosis include invasive techniques, such as intravascular ultrasound (IVUS), and non-invasive ultrasound examinations, such as magnetic resonance imaging (MRI), computerized tomography (CT), and positron emission tomography (PET) (Sarraju & Nissen, 2024). MRI uses multiple contrast-weighted images, including post-contrast T1-weighted images, to identify LRNCs and provides effective information on the volume and area of LRNCs (Cai et al., 2002). 18F-FDG PET/CT is often used in conjunction with MRI to assist in the detection and quantification of inflammatory activation within plaques (Yu et al., 2024). Carotid plaque CT studies have also confirmed that the composition of carotid atherosclerotic plaques determined by CT, such as the presence of a large LRNC, accurately reflects the plaque composition defined by histological examination (Wintermark et al., 2008). With the advancement of technology, the presence and size of the lipid core can be assessed using various detection methods, creating opportunities for in-depth research.

Over the past two decades, numerous studies have documented the presence and size of lipid cores in carotid plaques, as well as the incidence of major cardiovascular and cerebrovascular events. The objective of this study was to conduct a systematic review of all studies on the lipid core of carotid plaques and major cardiovascular and cerebrovascular events. Additionally, we performed a meta-analysis of previously reported results to explore the cumulative incidence of MACCEs in patients with lipid-core-containing carotid plaques, whether the lipid core is a risk factor for cardiovascular and cerebrovascular events, and whether the size of the lipid core (relative and absolute size) affects the occurrence of MACCEs, thereby providing a comprehensive overview of the relationship between the two.

## Materials & Methods

We conducted a systematic review and meta-analysis in accordance with the Preferred Reporting Items for Systematic Reviews and Meta-Analyses (PRISMA) guidelines and registered the study on the PROSPERO website (registration number: CRD42024611353).

### Eligibility criteria

We focused on the relationship between MACCEs and LRNCs in carotid plaques. Accordingly, we included observational studies, such as cohort studies, case-control studies, and cross-sectional studies. In accordance with the guidelines from the US Food and Drug Administration (FDA) in 2008 and the European Medicines Agency (EMA) in 2012, and considering the actual impact of LRNCs on cardiovascular and cerebrovascular events, we defined MACCEs as cardiac death, myocardial infarction, and ischemic stroke (Sharma et al., 2020). To ensure the rigor of our study, we excluded studies on cerebral infarction that were not explicitly identified as ischemic stroke, as well as those that, despite being identified as ischemic stroke, did not meet the World Health Organization’s definition of ischemic stroke, such as studies with ischemic symptoms lasting less than 24 h. Ipsilateral stroke was defined as an ischemic stroke occurring in the cerebral hemisphere supplied by the carotid artery containing the index LRNC. Strokes occurring in the hemisphere contralateral to the carotid plaque with LRNC were classified as contralateral stroke. This definition was applied consistently across all analyses involving stroke laterality. The specific inclusion criteria were as follows: (1) adults aged 18 years or older with carotid atherosclerosis; (2) detailed assessment of at least one aspect of the presence or size of LRNCs; (3) documentation of MACCEs (including cardiac death, myocardial infarction, and ischemic stroke); (4) no surgical treatment, such as carotid endarterectomy (CEA) or carotid artery stenting (CAS). We also excluded duplicate publications, studies with incomplete data, and those for which full-text articles were unavailable. The primary outcome of this review was the overall incidence of MACCEs or the incidence of at least one type of MACCE. The included studies should report at least one of the above outcomes. For studies contributing to the analysis of cumulative incidence (*i.e.,* assessing the risk of future MACCEs based on baseline LRNC status), we explicitly verified and required that the imaging assessment of the LRNC preceded all recorded outcome events. Studies where event ascertainment occurred concurrently with or prior to imaging were categorized separately and analyzed under different objectives (*e.g.*, plaque characteristics in patients with MACCEs).

Given the diversity and critical role of MRI in identifying LRNC components, we specified that MRI analyses in included studies must incorporate T2-weighted imaging (T2WI) sequences or other imaging sequences validated in the literature for the accurate measurement of LRNC (Kassem et al., 2020; Benson et al., 2023). The use of contrast agents and specific segmentation methods were not mandated, provided that the core imaging protocol met the above criterion for LRNC characterization.

### Cohort deduplication

When multiple publications from the same cohort study were identified, we selected the publication providing the most comprehensive data on our pre-specified outcomes using a pre-defined hierarchy: (1) completeness of MACCEs endpoint reporting, (2) duration of follow-up, (3) detail of LRNC quantification. Only one publication per unique patient cohort was included in any single meta-analysis.

### Information sources

We searched the PubMed, Embase, Web of Science, and Cochrane databases for all citations from inception to March 1, 2026.

### Search strategy

We identified relevant studies using the search strategy “A AND B AND C” in combination with MeSH terms and free-text words (A: “major adverse cardiovascular and cerebrovascular events” OR “cerebrovascular disease” OR “cardiovascular disease” OR “cardiac death” OR “myocardial infarction” OR “ischemic stroke”; B: “lipid-rich necrotic core”; C: “carotid atherosclerosis”). The complete search strings for each database are provided in Supplementary Material S1. In addition, we manually reviewed the reference lists of all included studies to identify any additional relevant studies that may have been missed.

### Selection process

The records obtained from the database search using the aforementioned strategy were imported into NoteExpress software (3.7.0.9296 version) for deduplication. Subsequently, two independent reviewers (YQ Sun and BR Zhang) screened the studies based on their titles and abstracts. In cases of disagreement, a third reviewer (QH Fan) made the final decision. The studies that passed the initial screening were then reviewed in full text by two independent reviewers (YQ Sun and LY Hu) to determine their eligibility. Any discrepancies were resolved by a third reviewer (SX Wu).

### Data collection

Data extraction from the included studies was conducted independently by two researchers (YQ Sun and QH Zhang), employing a pre-specified form within Microsoft Excel (2019 version). Resolution of any differing opinions was facilitated by a third investigator (SX Wu).

The extracted information included the basic characteristics of the studies (title, first author, year of publication, study type, data sources, research unit, sample size, diseases concerned in MACCEs and their diagnostic criteria, handle of missing data), characteristics of participants (gender, mean age, strain type, confounding assessed, follow-up period, inclusion and exclusion criteria, carotid concerned, case of MACCEs, case of LRNC in carotid plaques, imaging modality and retest result, response rates), and data of the results (mean LRNC area, max LRNC volume, percent LRNC volume in carotid plaque, OR and 95% CI for associated factors, timing of the measurement).

For cohorts reporting multiple effect estimates, we selected one primary effect size per analysis based on a pre-specified hierarchy prioritizing adjusted over unadjusted estimates, and hazard ratios over odds ratios when available. For every study that reported an adjusted effect estimate (*e.g.*, hazard ratio, odds ratio), we systematically extracted all covariates that were included in the adjustment model. These are presented in full detail in Supplementary Material S2. We recognized that LRNC area (mm^2^), volume (mm^3^), and proportion (%) represent fundamentally different dimensions of plaque composition (Table 1). To be specific, LRNC area (measured in mm^2^) refers to the cross-sectional area of the lipid-rich necrotic core within a single slice of the carotid plaque. LRNC volume (measured in mm^3^) refers to the total volume of the lipid-rich necrotic core across all slices of the carotid plaque. Percentage LRNC volume (measured as %) refers to the proportion of the total carotid plaque volume occupied by the lipid-rich necrotic core (*i.e.,* (LRNC volume/total plaque volume) ×100). Therefore, we conducted separate meta-analyses for each measurement type and did not pool estimate across different measurement dimensions. This approach ensures that all pooled estimates within a given meta-analysis are clinically and methodologically homogeneous.

**Table 1:** Definitions of units.

Units	Definitions
Area	The cross- sectional area of the LRNC, measured in mm^2^ on the slice showing the maximum plaque burden.
Volume	The total three- dimensional volume of the LRNC within the plaque, measured in mm^3^.
Proportion	The volume of the LRNC expressed as a percentage of the total vessel- wall volume (LRNC volume/vessel- wall volume ×100%).

For missing information (such as 95% confidence intervals and standard deviations), we used the Cochrane-recommended Excel conversion template to estimate values from *p*-values. For standard deviations requiring pooling, we applied the conversion formula for calculation. The detailed derivation process is provided in Supplementary Material S3.

### Risk of bias assessment

Two authors (YQ Sun and BR Zhang) will independently assess the quality of the studies, with results recorded in a pre-designed data extraction form using Excel software (version 2019). Any disagreements will be resolved by a third reviewer (QH Zhang).

The Agency for Healthcare Research and Quality (AHRQ) criteria, encompassing 11 items, will be applied to evaluate cross-sectional studies. Each item answered “yes” contributes one point. Studies scoring 8–11, 4–7, or 0–3 points are designated high, medium, or low quality, respectively. Cohort and case-control study quality will be assessed using the Newcastle-Ottawa Scale (NOS), which includes eight core items and one supplementary item. Similarly, one point is granted per “yes” answer. High-quality studies achieve 7–9 points, medium quality 4–6 points, and low quality 1–3 points. Sample size weighting will be applied to all included studies to determine both the item-specific overall scores and the total score aggregating all items.

### Sensitivity analysis

To evaluate the stability of the pooled estimates and identify potential sources of heterogeneity, we performed a leave-one-out sensitivity analysis by iteratively excluding individual studies and recalculating the combined effect sizes. The impact of each study was quantified by comparing the changes in the pooled cumulative incidence before and after exclusion. Additionally, we conducted sensitivity analyses using different statistical models (fixed-effect *vs.* random-effect) and heterogeneity estimation methods (Cochran’s Q test and I^2^ statistic).

### Publication bias analysis

We systematically assessed publication bias using both visual and statistical methods. We evaluated funnel plot asymmetry using Egger’s linear regression test and Begg’s rank correlation test, with a *p*-value < 0.05 indicating significant asymmetry. A tiered approach was adopted for assessing funnel plot asymmetry. For meta-analyses comprising at least five studies, publication bias was assessed quantitatively using Egger’s linear regression test. For meta-analyses with fewer than five studies, quantitative tests were deemed statistically inappropriate due to insufficient power; thus, only visual inspection of the funnel plot was performed, with no statistical inference drawn.

### Subgroup analysis

We conducted prespecified subgroup analyses to explore potential effect modifiers and sources of heterogeneity. Studies were stratified by the following factors: study design (cross-sectional studies or else), imaging modality (MRI imaging or else), and stroke subtype (general ischemic stroke, ESUS or else). We assessed heterogeneity between subgroups using Cochran’s Q test and I^2^ statistic and quantified the proportion of variance explained by subgroup variables using either a fixed-effect or random-effects model.

### GRADE assessment of evidence certainty

We used the Grading of Recommendations Assessment, Development and Evaluation (GRADE) approach to assess the certainty of evidence for key outcomes. Two reviewers (YQS and BRZ) independently evaluated each outcome for risk of bias, inconsistency, indirectness, imprecision, and publication bias. In accordance with GRADE guidance, the baseline certainty for evidence derived from observational studies is low. The certainty was then downgraded or upgraded (*e.g.*, for a large magnitude of effect) based on these criteria. The assessments were performed using the GRADEpro GDT online tool (https://www.gradepro.org/), provided by Cochrane, which facilitates the process by providing a structured interface to document judgments. Discrepancies were resolved through discussion or by a third reviewer (SXW). The overall certainty of evidence was categorized as high, moderate, low, or very low. The GRADE evidence profile is provided in Supplementary Material S4.

### Statistical analyses

Data collected in Excel will be imported into R software (4.2.3 version) for analysis. The following packages will be used for analysis and visualization: gridExtra, meta, robvis, ggplot2, tidyverse, and reshape2. Categorical variables will be summarized using hazard ratios (HR), odds ratios (OR) and their 95% confidence intervals (CI). Continuous variables will be analyzed using mean differences (MD) ± standard deviations (SD) and presented as MD with 95% CI. For overall pooled estimates synthesizing data across diverse study designs and populations, we employed the random-effects model as the primary method. This accounts for between-study variance and provides a more generalizable estimate for heterogeneous data. However, our analysis involved detailed stratification into specific, clinically homogeneous comparisons (*e.g.*, ipsilateral *vs.* contralateral plaques, or specific LRNC metrics). Many of these finely-stratified meta-analyses incorporated a limited number of studies (typically *n* ≤ 3). In such cases, the random-effects model cannot reliably estimate the between-study variance component, potentially leading to less robust results (Borenstein et al., 2010; Maitra, 2025). Therefore, for these specific subgroup and outcome analyses, we utilized the common-effect model, which is statistically appropriate when combining a small number of studies under a common hypothesis. The model used for each specific forest plot is explicitly stated in the corresponding figure legend or results text.

## Results

**Figure 1:**
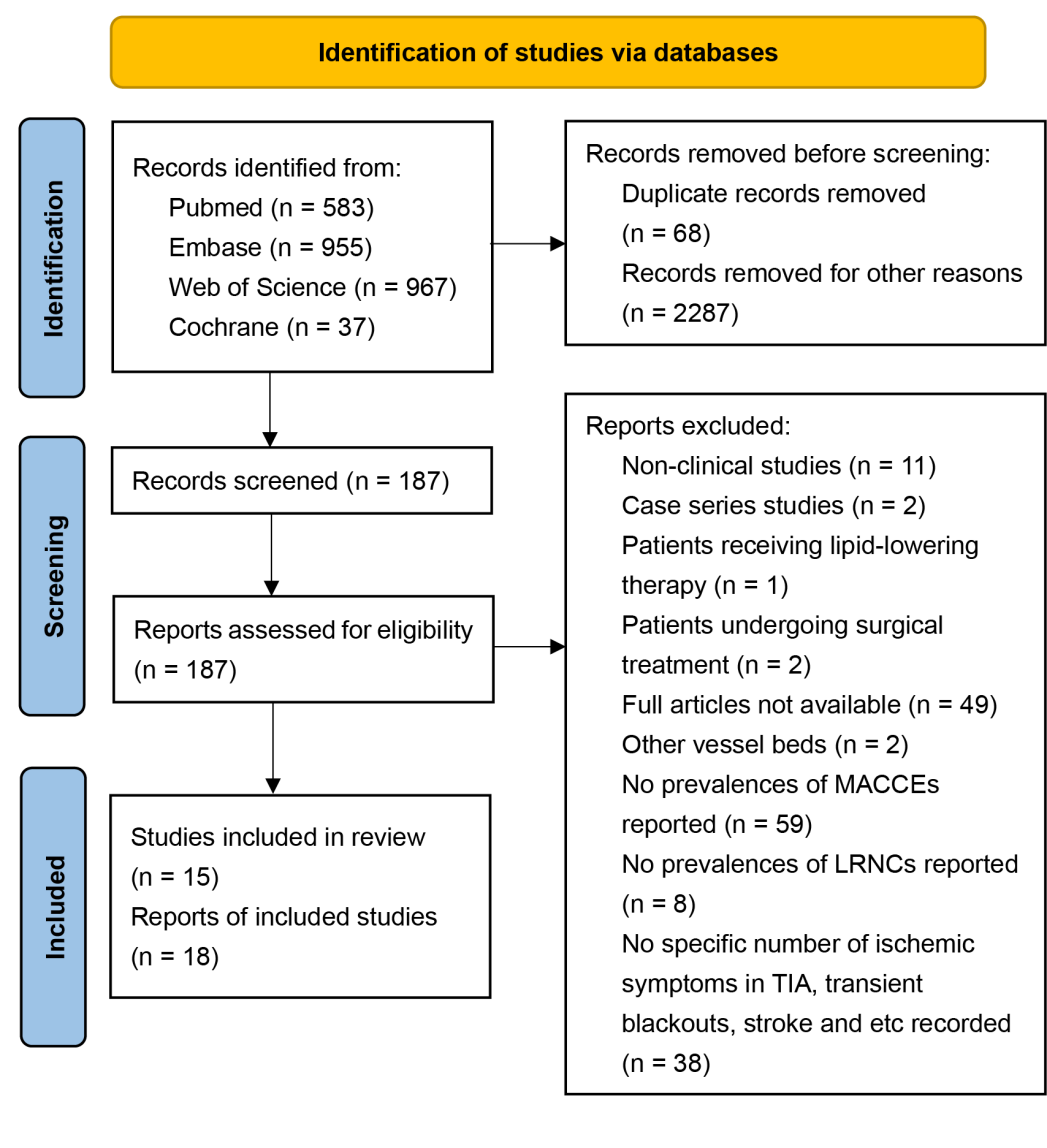
Flowchart of identification and selection of included articles *via* databases.

We initially identified 2,542 citations from four major databases: Embase (*n* = 955), PubMed (*n* = 583), Web of Science (*n* =967), and Cochrane (*n* = 37). After removing 68 duplicate records, we screened the remaining 2,474 citations by title and abstract, excluding 2,287 that were not relevant to our study. This left us with 184 full-text articles for detailed review. Ultimately, 15 articles met our inclusion criteria. The primary reason for exclusion was the absence of reported MACCEs (*n* = 56) (Fig. 1).

### Study characteristics

A total of 18 studies from 15 eligible articles (Dalager et al., 2007; Magge et al., 2013; Grimm et al., 2013; Hyafil et al., 2016; Saam et al., 2016; Xu et al., 2016; Che et al., 2021; Giannotti et al., 2021; Brunner et al., 2021; Bos et al., 2021; Song et al., 2021; Guo et al., 2022; Van der Toorn et al., 2022; Lu et al., 2022; Tang et al., 2023) were included in the meta-analysis, comprising 5,682 patients and 6,257 carotid plaques. Three articles reported on two cohorts each. The studies were published between 2007 and 2023, with sample sizes ranging from 14 to 1,349 patients. Among the 5,682 patients, 2,779 were male, with a mean age of 65.7 years. Follow-up durations varied from 3.1 months to 126 months, with a mean follow-up of 52.5 months. The studies covered a range of MACCEs, including stroke (*n* = 17), cardiac death (*n* = 2), myocardial infarction (*n* = 1), recurrent stroke (*n* = 3), and embolic stroke of undetermined source (ESUS) (*n* = 2). The research designs included case-control studies (*n* = 1), cross-sectional studies (*n* = 7), and cohort studies (*n* = 7). Imaging modalities used were CT (*n* = 5), histological staining (*n* = 1), and MRI (*n* = 12). The studies assessed various characteristics of LRNCs, including prevalence and American Heart Association (AHA) lesion type distribution. LRNC size was evaluated in terms of absolute size (mean and maximum LRNC area and volume) and relative size (% volume of LRNC and plaque components as a percentage of the vessel wall). The studies were categorized into three groups based on their objectives: (1) the incidence of cardiovascular and cerebrovascular events in patients with carotid plaques (*n* = 6), aiming to assess the risk of future events. To ensure proper causal inference, we included only studies in which LRNC assessment *via* imaging preceded the occurrence of any MACCEs. We systematically verified the timing of assessment for each included study; (2) plaque characteristics in patients with cardiovascular and cerebrovascular events (*n* = 5), focusing on plaque status at the time of the event; and (3) comparison of ipsilateral and contralateral plaques in stroke patients (*n* = 7), investigating whether stroke hemisphere is associated with differences in carotid plaque status. A detailed overview of the included studies is provided in Supplementary Material S5.

### Risk of bias in studies

**Figure 2:**
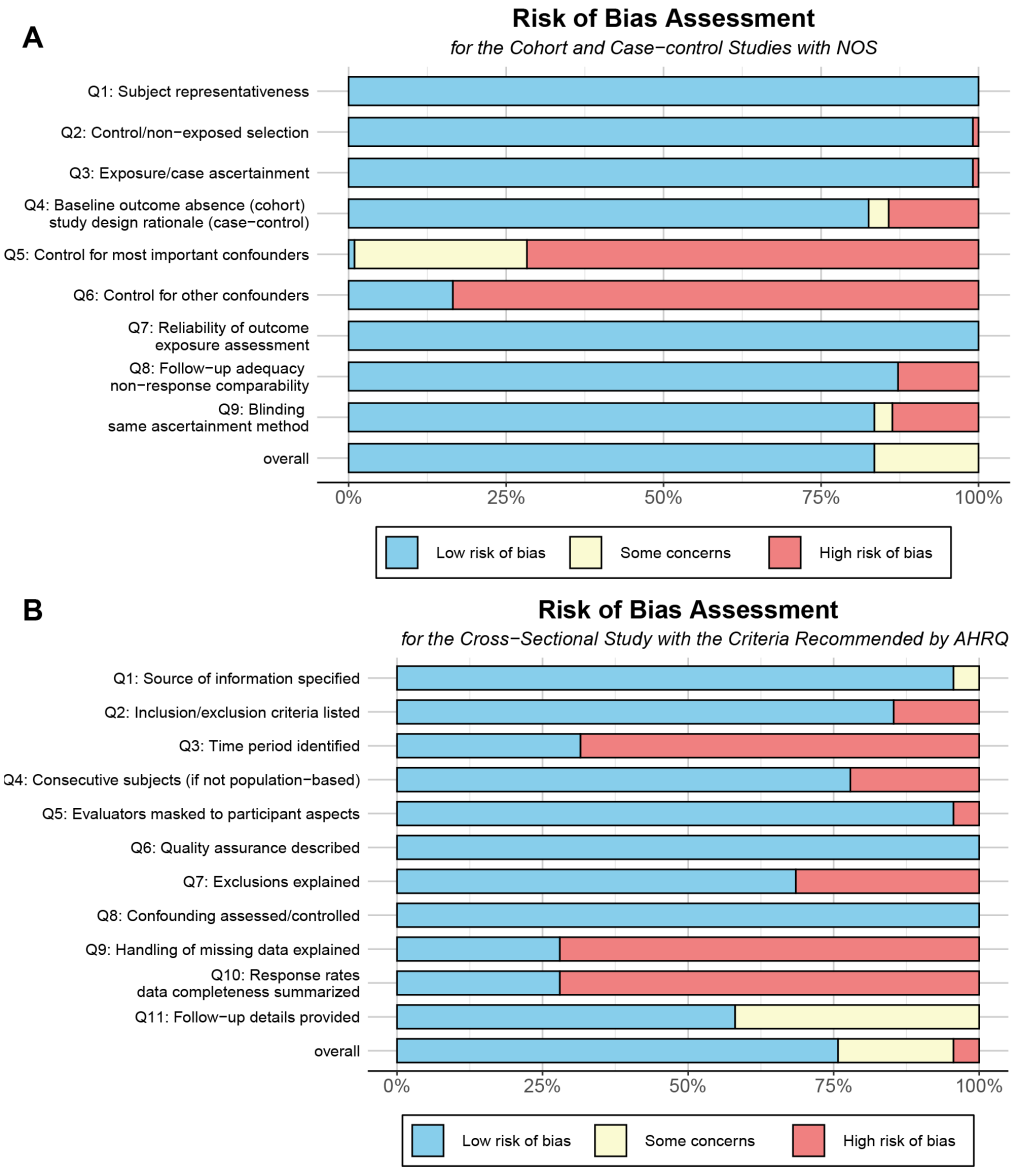
Risk of bias assessment. (A) The risk of bias assessment for the cohort and case-control studies with NOS. (B) The risk of bias assessment for the cross-sectional studies with the criteria recommended by AHRQ.

The risk of bias assessment for each study is provided in Supplementary Material S6. The results indicate that among the included studies, three were classified as low quality, five as high quality, and seven as medium quality. The AHRQ scores for seven studies ranged from three to nine, with a mean score of seven. The included studies generally scored lower on items related to handling of confounding factors and missing data. The NOS scores for the eight included studies ranged from four to seven, with a mean score of 5.9. These studies also scored lower on controlling for confounding factors (Fig. 2).

### Certainty of evidence (GRADE)

The certainty of evidence for each outcome was assessed using the GRADE approach (Supplementary Material S4). The cumulative incidence of MACCEs in patients with LRNC was rated as moderate certainty, downgraded due to inconsistency (high heterogeneity). The proportion of ipsilateral stroke in patients with LRNC was rated as high certainty. The associations between LRNC size (mean volume, max area, and % volume) and MACCEs were rated as high certainty, except for the % LRNC volume outcome, which was downgraded to moderate certainty due to imprecision.

### The cumulative incidence of MACCEs

**Figure 3:**
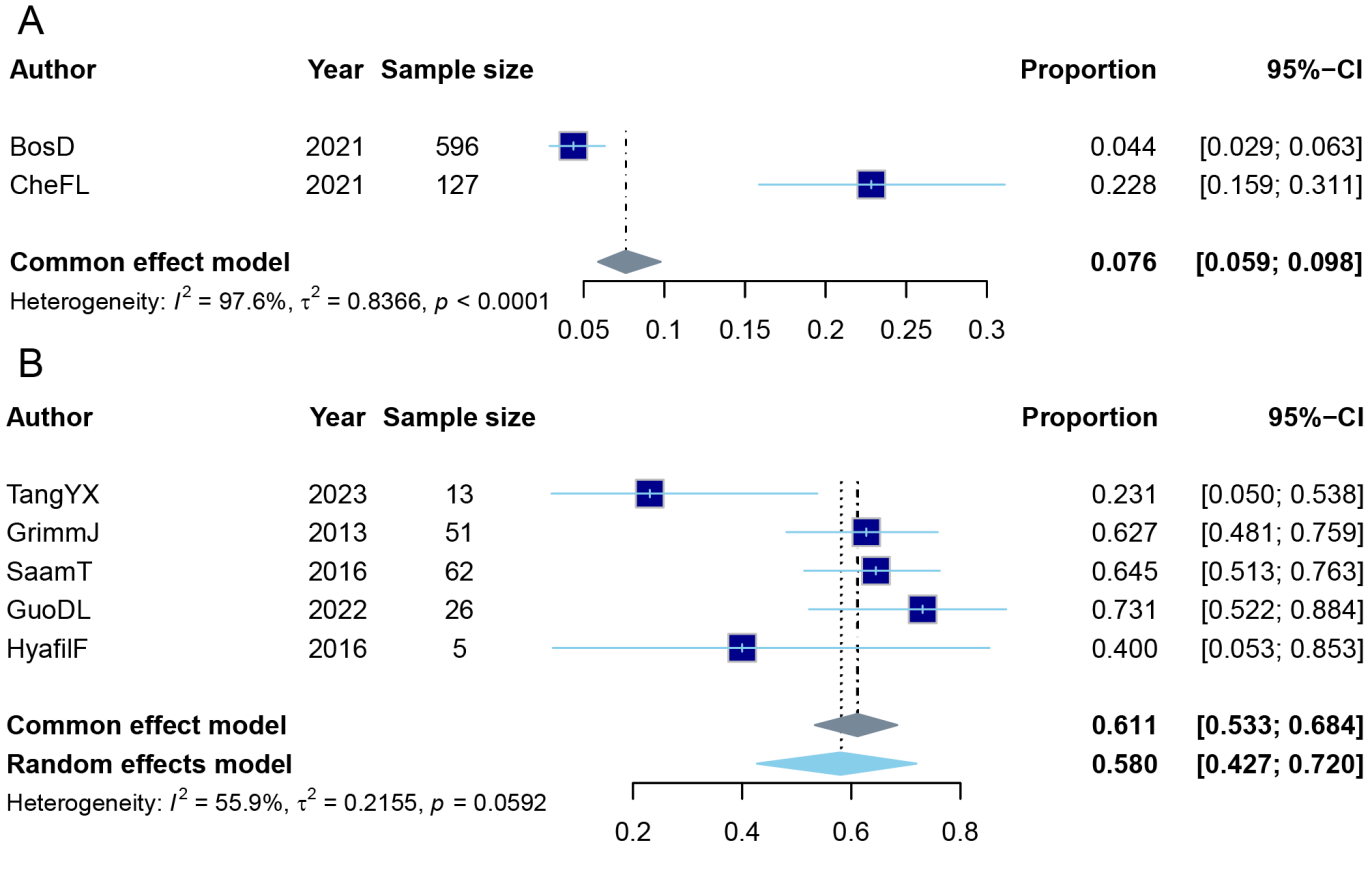
Meta-analysis on the cumulative incidence of MACCEs. (A) The result of meta-analysis on the cumulative incidence of MACCEs in patients with LRNC of carotid plaques. (B) The result of meta-analysis on stroke laterality: the rate of ipsilateral stroke in patients with LRNC of carotid plaques. Ipsilateral stroke was defined as an ischemic stroke occurring in the cerebral hemisphere supplied by the carotid artery containing the index LRNC. The dark blue box represents the study. The sky-blue diamond is the result of the random-effect meta-analysis. The gray diamond is the result of the common-effect meta-analysis.

The meta-analysis included two studies investigating the cumulative incidence of MACCEs in patients with carotid plaques. All studies were longitudinal cohorts where LRNC assessment preceded event ascertainment. The results showed that in individuals with lipid-core-containing carotid plaques, the cumulative incidence of MACCEs was approximately 7.6% (95% CI [0.059–0.098], common effect model, *k* = 2) (Fig. 3A) and funnel plot visual inspection did not reveal substantial asymmetry (Supplementary Material S7). The combined mean follow-up duration for this analysis was 32.2 months (range: 3.1 to 61.2 months). The pooled analysis of two cohort studies, comprising a total of 3,072.4 person-years of follow-up, recorded 55 MACCEs. This yielded an incidence rate of 17.9 events per 1,000 person-years (95% CI [13.5–23.4]). The individual study estimates demonstrated substantial variability (*I*^2^ = 97.6%, *p* < 0.01). Bos et al. (2021), with a sample size of 596 patients, reported a proportion of 0.044 (95% CI [0.029–0.063]). In contrast, Che et al. (2021), with a smaller sample of 127 patients, reported a considerably higher proportion of 0.228 (95% CI [0.159–0.311]). In the stroke laterality analysis, which included five studies comparing ipsilateral and contralateral plaques in stroke patients, we found that when a stroke occurred, the probability of it occurring on the same side as a lipid-core-containing plaque was approximately 58.0% (95% CI [0.427–0.720], random effect model, prediction interval [0.2254–0.8679], *k* = 5) (Fig. 3B). This estimate of 58.0% is presented as the primary result in the Abstract and throughout the manuscript.

Given that the heterogeneity in the second study could still be reduced (*I*^2^ = 55.9%, *p* = 0.0592), we conducted sensitivity analyses, publication bias analyses, and subgroup analyses to further decrease heterogeneity. The sensitivity analysis revealed that excluding the study by Tang et al. (2023) significantly reduced heterogeneity, resulting in a final cumulative incidence of 64.6% (95% CI [0.564–0.720], random effect model, prediction interval [0.5116–0.7605], *k* = 4) (I^2^ = 0%, *p* = 0.5521) (Supplementary Material S8A, Supplementary Material S8E). The publication bias analysis showed no evidence of publication bias among the included studies (*t* =  − 1.46, *df* = 3, *p*-value = 0.2406, Supplementary Material S9). The subgroup analyses indicated that stratifying the studies by imaging modality (MRI), stroke type (general ischemic stroke, excluding ESUS), and study design (cross-sectional studies) each reduced heterogeneity. The resulting ipsilateral stroke rate were 0.532 (95% CI [0.348–0.707], random effect model, *k* = 4), 0.532 (95% CI [0.348–0.707], random effect model, *k* = 4), and 0.646 (95% CI [0.538–0.742], random effect model, *k* = 3), respectively (Supplementary Material S8B, Supplementary Material S8D).

### Risk factor

**Figure 4:**
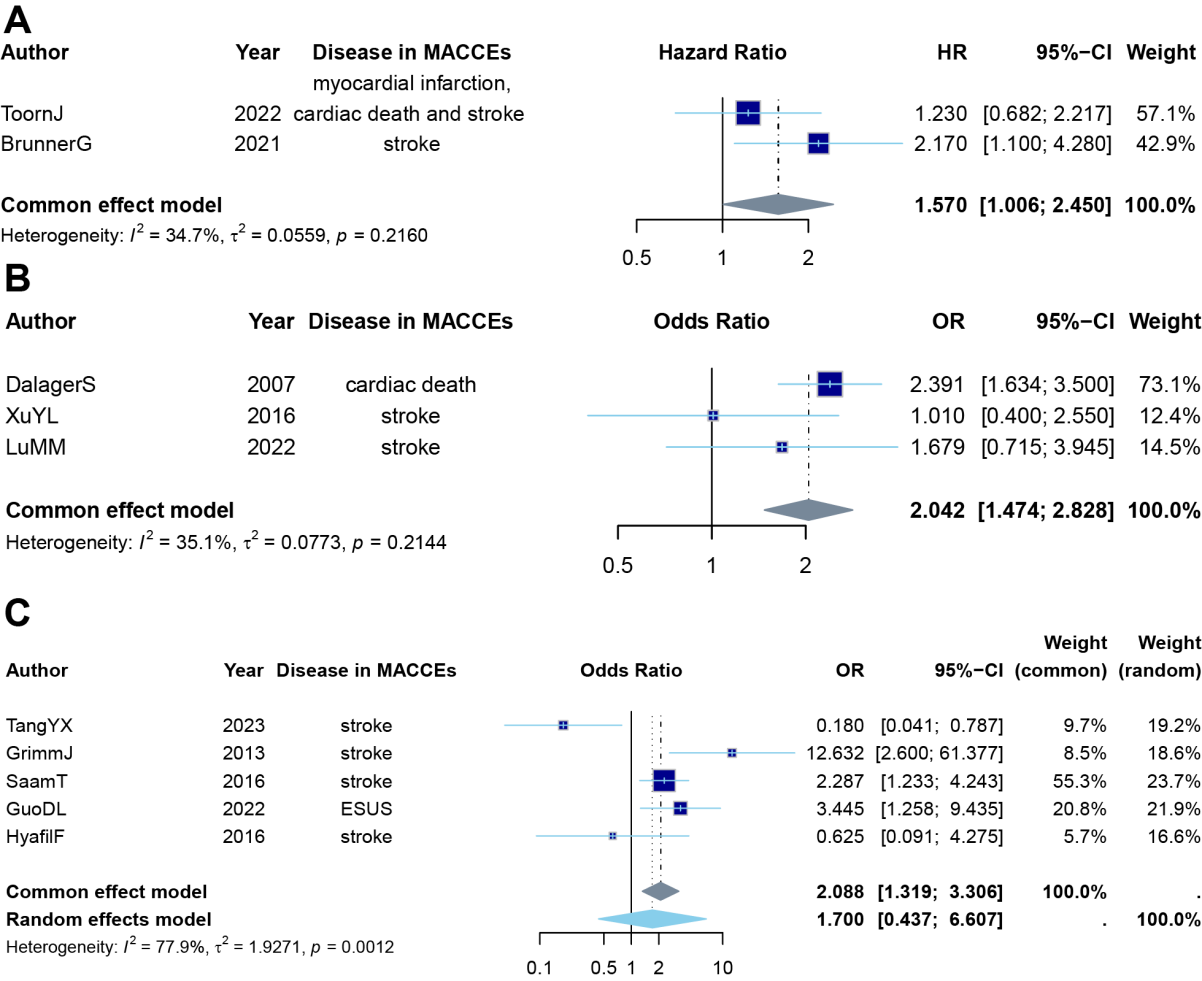
Meta-analysis on the MACCEs risk ratio. (A) Meta-analysis of the hazard ratio for MACCEs in patients with LRNC in carotid plaques from studies investigating the cumulative incidence of MACCEs in patients with carotid plaques. (B) Meta-analysis of the odds ratio for MACCEs in patients with LRNC in carotid plaques from studies examining the prevalence of LRNCs in patients with MACCEs. Ipsilateral stroke was defined as an ischemic stroke occurring in the cerebral hemisphere supplied by the carotid artery containing the index LRNC. (C) Meta-analysis of the odds ratio for stroke laterality: the likelihood of stroke occurring on the ipsilateral side in patients with LRNC in carotid plaques. The size of the dark blue box, representing the beta coefficient, is proportional to the study weight. The gray diamond indicates the result of the common-effect meta-analysis.

The meta-analysis included five studies comparing ipsilateral and contralateral plaques in stroke patients, where ipsilateral stroke was defined as an ischemic stroke occurring in the cerebral hemisphere supplied by the carotid artery containing the index LRNC, three studies investigating the cumulative incidence of cardiovascular and cerebrovascular events in patients with carotid plaques, and three studies examining the prevalence of LRNCs in plaques of patients with MACCEs. The results showed that in patients with carotid plaques, the presence of an LRNC is a risk factor for MACCEs (HR: 1.570, 95% CI [1.006–2.450], common effect model, *k* = 2) (Fig. 4A) and funnel plot visual inspection did not reveal substantial asymmetry (Supplementary Material S10). For this pooled estimate, all contributing studies reported adjusted effect sizes (see Supplementary Material S2 for covariates). Similarly, in patients experiencing MACCEs, the presence of an LRNC in carotid plaques is a risk factor for MACCEs (OR: 2.042, 95% CI [1.474–2.828], common effect model, *k* = 3) (Fig. 4B) and funnel plot visual inspection did not reveal substantial asymmetry (Supplementary Material S11). For this pooled estimate, two of the three contributing studies reported adjusted effect sizes (see Supplementary Material S2 for covariates). Given the small number of studies, a sensitivity analysis restricted to only adjusted estimates would be statistically underpowered. In the stroke laterality analysis, however, strokes tended to occur ipsilaterally to LRNC in the same cerebral hemisphere, but showed no significant difference compared to the contralateral side (OR: 1.7, 95% CI [0.437–6.607], random effect model, *k* = 5, prediction interval [0.0229–126.2175]) (Fig. 4C) without obvious publication bias (*t* =  − 0.46, *df* = 3, *p*-value = 0.6790, Supplementary Material S12). For this pooled estimate, no contributing studies reported adjusted effect sizes (see Supplementary Material S2 for covariates). To assess the impact of imaging methodology on the prior finding, we performed exploratory subgroup analyses. The result remained consistent when stratifying studies by imaging modality (OR: 1.383, 95% CI [0.241–7.918], random effect model, *k* = 4, Supplementary Material S13) and, within the MRI subset, by use of contrast agent (OR: 2.744, 95% CI [0.621–12.130], random effect model, *k* = 3, Supplementary Material S14). The detailed results of these subgroup analyses are presented in Supplementary Material S15.

Some studies classified plaques according to the American Heart Association (AHA) criteria, where type IV plaques have a large lipid core and type V plaques have a thick fibrous cap over the lipid core; both are often collectively referred to as “lipid-core plaques” (Cai et al., 2002). We have provided the detailed AHA classification and LRNC composition in the Supplementary Material S16. A meta-analysis of these studies revealed that, compared with strokes on the opposite side of the lipid core, ipsilateral stroke associated with lipid-core plaques classified by AHA criteria is not a risk factor, although one study indicated that lipid-core plaques are a risk factor for sudden cardiac death (Supplementary Material S17).

### The correlation between mean LRNC volume and MACCEs

**Figure 5:**
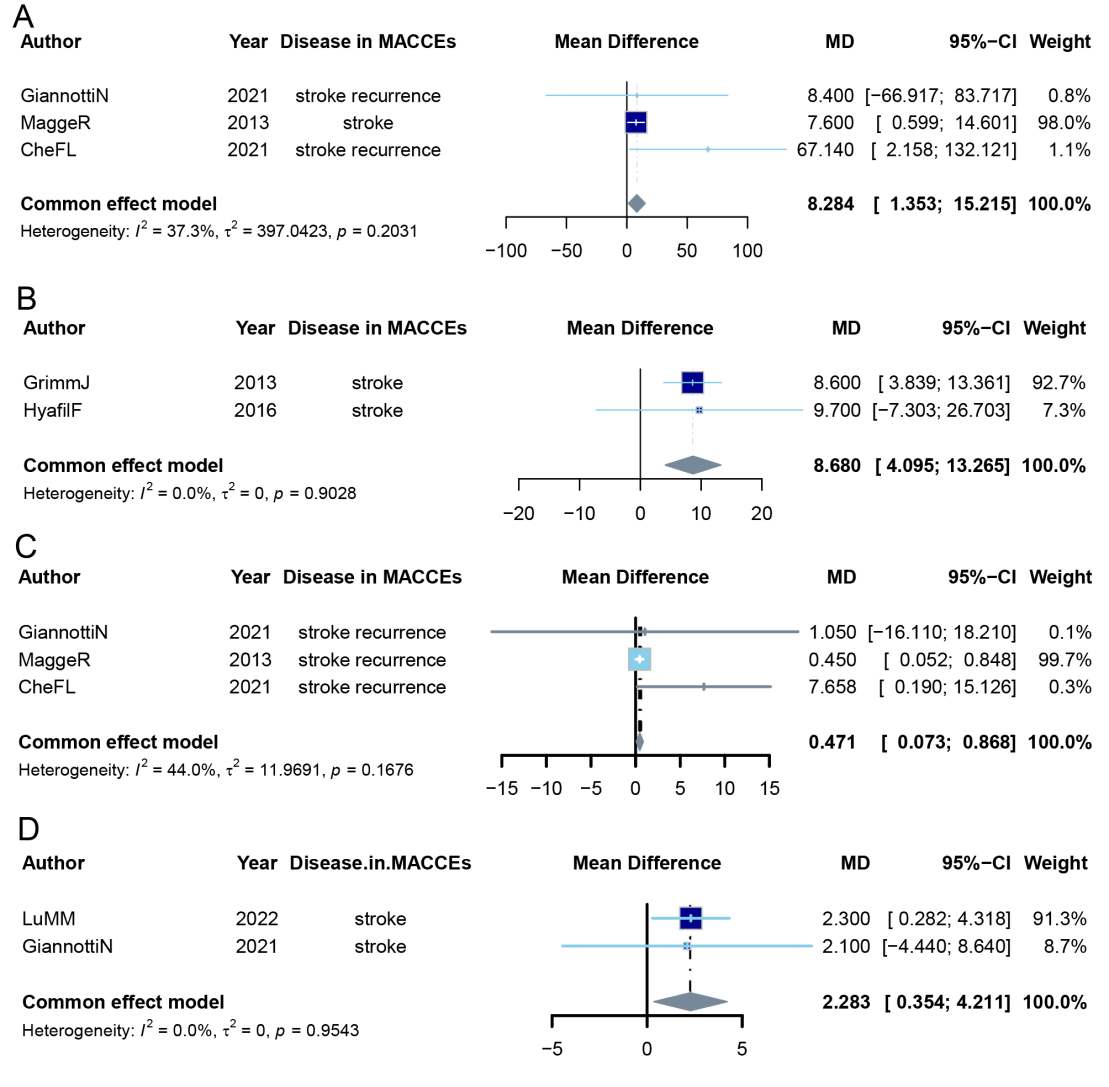
Meta-analysis on the LRNC size. (A) The result of meta-analysis on the correlation between mean LRNC volume (mm^3^) and MACCEs. (B) The result of meta-analysis on the correlation between max LRNC area (mm^2^) and stroke laterality. Ipsilateral stroke was defined as an ischemic stroke occurring in the cerebral hemisphere supplied by the carotid artery containing the index LRNC. (C) The result of meta-analysis on the correlation between % LRNC volume (%) and MACCEs from studies examining the cumulative incidence of MACCEs in patients with plaques. (D) The result of meta-analysis on correlation between % LRNC volume (%) and MACCEs from studies examining the prevalence of LRNCs in patients with MACCEs. The size of the dark blue box, which represents the beta, is proportional to the weight of the study. The gray diamond is the result of the common-effect meta-analysis.

The meta-analysis included three studies investigating the cumulative incidence of cardiovascular and cerebrovascular events in patients with carotid plaques, two studies examining the average volume of LRNCs in plaques of patients experiencing MACCEs, and in the stroke laterality analysis, two studies comparing the volume of LRNCs in ipsilateral and contralateral plaques in stroke patients. The results showed that, compared with patients without MACCEs, those with MACCEs had larger mean LRNC volumes (in mm^3^) (MD: 8.284 mm^3^, 95% CI [1.353–15.215], common effect model, *k* = 3) (Fig. 5A) and funnel plot visual inspection did not reveal substantial asymmetry (Supplementary Material S18). In patients with stroke, the average LRNC volume in ipsilateral plaques was larger than that in contralateral plaques, although the difference was not statistically significant (Supplementary Material S19). Similarly, when comparing patients with MACCEs to those with other ischemic symptoms, the mean LRNC volume was larger in patients with MACCEs, but the difference was not statistically significant (Supplementary Material S20).

### The correlation between maximum LRNC area and MACCEs

Due to the limited number of available studies, our meta-analysis included two studies comparing the area of LRNCs in ipsilateral and contralateral plaques in stroke patients. The results indicated that, those ipsilateral plaques had larger maximum LRNC areas (in mm^2^) (MD: 8.68 mm^2^, 95% CI [4.095–13.265], common effect model, *k* = 2) (Fig. 5B) and funnel plot visual inspection did not reveal substantial asymmetry (Supplementary Material S21).

### The correlation between proportion of LRNC volume in the vessel wall and MACCEs

The meta-analysis included three studies investigating the cumulative incidence of MACCEs in patients with carotid plaques, two studies examining the average volume of LRNCs in plaques of patients experiencing MACCEs, and two studies comparing the volume of LRNCs in ipsilateral and contralateral plaques in stroke patients. Compared with patients without MACCEs, those with a higher proportion of LRNC volume in the plaque (in %) were at greater risk of future MACCEs (MD: 0.471%, 95% CI [0.073–0.868], common effect model, *k* = 3) (Fig. 5C) and funnel plot visual inspection did not reveal substantial asymmetry (Supplementary Material S22). Additionally, compared with patients with cerebral ischemic symptoms, those experiencing MACCEs had a higher proportion of LRNC volume in the plaque (in %) (MD: 2.283%, 95% CI [0.354–4.211], common effect model, *k* = 2) (Fig. 5D) and funnel plot visual inspection did not reveal substantial asymmetry (Supplementary Material S23). No significant difference was found in the proportion of LRNC volume in the vessel wall between ipsilateral and contralateral plaques in patients with stroke (Supplementary Material S24).

## Discussion

### Summary of the main findings

In our meta-analysis, we included 15 articles encompassing 18 studies involving 5,682 patients and 6,257 carotid plaques. These studies were categorized into three groups based on their research objectives: (1) incidence of MACCEs in patients with plaques, (2) plaque characteristics in patients with MACCEs, and (3) comparison of ipsilateral and contralateral plaques in stroke patients. The results indicated that the incidence of MACCEs over a mean follow-up of ∼32.2 months (range 3.1–61.2 months) was approximately 7.6% in individuals with lipid-rich carotid plaques. Previous meta-analyses have reported an annual incidence rate of ischemic events in patients with LRNC of approximately 5–8% (Mono et al., 2012). Furthermore, the probability of stroke occurrence on the same side as the lipid-rich plaque was about 58.0%. Our findings also revealed that the presence of a lipid core in carotid plaques is a risk factor for MACCEs, consistent with prior studies (Rizvi et al., 2020).

### Interpretation of the results

We observed substantial heterogeneity in the cumulative incidence of MACCEs among studies involving individuals with lipid-rich carotid plaques. The cumulative incidence varied significantly across studies, which we attribute to several factors. First, the limited number of studies included in our analysis may have contributed to the observed heterogeneity. Second, studies with higher cumulative incidence included patients with recurrent ischemic stroke, which has been shown to be associated with LRNC that can increase the recurrence rate of cerebral ischemic events (Kwee et al., 2013). These factors are likely significant contributors to the heterogeneity observed in our meta-analysis.

When analyzing the proportion of stroke occurring on the same side as lipid-rich plaques, we found considerable variation, ranging from 23.1% to 73.1%, with moderate heterogeneity. This proportion did not systematically change with increasing sample size or study duration. Sensitivity analysis revealed that excluding one particular study significantly reduced heterogeneity, indicating that this study was a major source of heterogeneity. The proportion of stroke on the same side as lipid-rich plaques was 58.0% (95% CI [0.427–0.720]). The excluded study (Tang et al., 2023) recruited patients with plaques only on the ipsilateral or contralateral side of the stroke, whereas other studies included patients with plaques on both sides, which may have influenced the heterogeneity. Subgroup analysis showed that excluding patients with ESUS and conducting a meta-analysis only on patients with general ischemic stroke reduced heterogeneity. Similarly, meta-analyses restricted to MRI-detected results or cross-sectional studies also showed reduced heterogeneity. These findings suggest that the type of stroke and imaging methods used in the included studies are likely contributors to the observed moderate heterogeneity.

In our study, we identified the presence of a lipid core as a risk factor for MACCEs, which is consistent with current understanding (Ma et al., 2023). However, when plaques were classified according to the AHA classification—where Type IV denotes an atheroma with a confluent lipid-rich necrotic core, Type V a fibroatheroma (typically with a thicker fibrous cap overlying the LRNC), and Type VI a lesion with a thin or ruptured fibrous cap—the association between ‘lipid-core plaques’ and MACCEs was attenuated (see Supplementary Material S16 for detailed mapping of AHA categories to LRNC characteristics) (Cai et al., 2002). This discrepancy may arise from the fact that the AHA classification emphasizes morphological features of plaques (such as fibrous cap thickness, calcification, lipid content, and status), while the volume or spatial distribution of the lipid core may not be fully incorporated into the classification criteria. Moreover, although both Type IV and Type V plaques are categorized as lipid core plaques, Type V plaques, compared with Type IV plaques, have a thicker fibrous cap in addition to a large lipid core, which reduces the risk of rupture and consequently lowers the risk of stroke, thereby potentially affecting the overall results (Glagov et al., 1988). Moreover, AHA classes signal the presence and morphological type of an LRNC but do not quantify its absolute or relative burden. Consequently, plaques with similar AHA grades may harbor vastly different amounts of necrotic core, which our meta-analysis demonstrates is a critical determinant of risk. Additionally, despite the high sensitivity of MRI to fat tissue and its relatively high accuracy, different acquisition sequences can also affect the accuracy of plaque component identification, leading to misclassification of plaque types. For instance, Kawasaki et al. (2009) suggested that it is difficult to distinguish intraplaque hemorrhage from lipid components using T1-weighted imaging alone. Li et al. (2012) compared histological results with multi-contrast-weighted MRI results and found that 40% of AHA Type I-II plaques were misclassified as Type IV-V plaques, which could impact the results. In light of the limitations of the AHA classification, some scholars have attempted to establish new classification methods in recent years to better predict the risk of cardiovascular and cerebrovascular events. For example, Saba et al. (2024) established the Plaque-RADS category through expert consensus to better identify stroke risk.

Our finding that the presence of LRNC is a significant risk factor for MACCEs extends and refines the understanding derived from some landmark population-based studies. For instance, the prospective Rotterdam Study by Bos et al. (2021), which focused on initially asymptomatic individuals, reported that while IPH was a strong independent predictor, the mere presence of an LRNC was not significantly associated with future stroke or coronary heart disease. This apparent discrepancy may be reconciled by fundamental methodological distinctions. The Rotterdam Study assessed LRNC as a binary trait (present/absent), whereas our meta-analysis quantitatively synthesizes data on LRNC volume and proportion. Our results demonstrate that larger LRNC size, both in absolute and relative terms, is indeed associated with higher event risk. Therefore, our quantitative synthesis of LRNC burden (volume and proportion) helps reconcile apparent discrepancies with some prior population studies that treated LRNC as a binary trait (present/absent). This suggests that the quantitative burden of the lipid core, rather than its simple presence, may be the critical determinant of risk, especially in populations already manifesting clinical atherosclerosis. Therefore, our work underscores the importance of moving beyond qualitative assessment to standardized volumetric measurement of LRNC in risk stratification models. These results suggest that quantitative assessment of the lipid core may serve as an important indicator for risk stratification. However, it is important to note that, despite the general predictive value of lipid core size for MACCEs, our study found no significant difference in lipid core size between ipsilateral and contralateral plaques in stroke patients. On the one hand, plaque rupture, which may be more likely to occur due to changes in local blood flow shear stress, could lead to ischemic stroke rather than relying solely on differences in lipid core volume (Chen et al., 2024). On the other hand, atherosclerosis is a systemic condition and is not limited to a single carotid artery. The formation of lipid cores is associated with systemic lipid levels, such as low density lipoprotein (LDL) and apoB, which may explain why there is no significant difference in lipid core size between ipsilateral and contralateral plaques in stroke patients (Miceli et al., 2024; Borén, Packard & Binder, 2025). The contribution of LRNC to plaque vulnerability must be contextualized where it frequently co-occurs with other high-risk features, most notably IPH and a thin or ruptured fibrous cap. Empirical evidence strongly supports this co-occurrence. A recent imaging study specifically found a significant association between the presence of IPH and LRNC in carotid arteries, underscoring that these features are not isolated but often present together (Nguyen et al., 2026). Furthermore, a systematic review and meta-analysis on intracranial atherosclerosis concluded that the identification of a constellation of high-risk plaque characteristics—which includes components like LRNC—provides independent prognostic value for predicting stroke recurrence (Shi et al., 2025). Together, these studies suggest that LRNC may reflect, in part, the risk of the overarching high-risk plaque phenotype of which it is a key component.

### Strength and limitations

A strength of our study is that it is the first comprehensive assessment of the relationship between LRNC and MACCEs. We analyzed the relationship between LRNC and MACCEs from both qualitative and quantitative perspectives. First, we evaluated whether the presence of LRNC is indeed a risk factor for future MACCEs by assessing the risk of MACCEs in patients with LRNC. Second, we assessed the relationship between the size of the lipid core (both in absolute terms, such as area and volume, and in relative terms, such as the proportion of the plaque volume) and MACCEs. Additionally, by including both observational and case-control studies, we were able to explore bidirectional relationships. For example, comparing the characteristics of LRNC before and during an event provides key insights into the role of plaque composition in event prediction.

Limitations of our study include the relatively small number of studies included, with only 16 articles ultimately entering the meta-analysis. This number decreased further after categorizing the articles. The reason for this may be that the occurrence of MACCEs requires long-term follow-up, and our definition of MACCEs was stringent, excluding many studies that included transient ischemic attacks (TIAs), transient visual obscurations, and other ischemic symptoms. Thus, the limited long-term follow-up data may have underestimated the true association between LRNC progression and delayed MACCEs. Additionally, different quantification methods for LRNC (such as MRI, ultrasound, and histopathology) may introduce measurement bias. While we defined minimum imaging standards for LRNC identification by MRI and found consistent results across subgroups where analyzable, residual heterogeneity may still stem from unmeasured technical variations (*e.g.*, different MRI field strengths, segmentation software). The detailed imaging parameters of all studies are provided in Supplementary Material S15 for full transparency. Residual confounding factors (such as age and sex) could not be fully addressed (Derlin et al., 2015; Van Dam-Nolen et al., 2023). Additionally, while we employed a hierarchy prioritizing adjusted estimates, the number of studies reporting fully adjusted effect sizes for our primary outcomes was limited. Although we provide full transparency on the covariates adjusted for in each study (Supplementary Material S2), the variability in adjustment models precluded a formal quantitative assessment (*e.g.*, meta-regression) of their impact on the pooled estimates. This limited statistical power, particularly for subgroup and sensitivity analyses, and precluded the reliable use of more advanced quantitative techniques like meta-regression to formally assess moderators of heterogeneity (López-López et al., 2014). Furthermore, the independent prognostic value of the co-occurrence of LRNC with other high-risk features like intraplaque hemorrhage and thin fibrous cap could not be precisely quantified, as most primary studies did not perform analyses stratified by or adjusted for these co-pathologies.

The GRADE assessment indicated that the evidence supporting the association between LRNC presence/size and MACCEs is generally of moderate to high certainty, reinforcing the clinical relevance of our findings. However, the certainty was downgraded for some outcomes due to heterogeneity and imprecision, highlighting the need for more standardized and large-scale studies to enhance the robustness of these associations.

### Implications for clinical practice

Given that the presence and size of LRNC can serve as imaging biomarkers to identify high-risk patients for MACCEs, supplementing traditional risk factors such as the severity of stenosis, we propose that clinicians increase monitoring of LRNC to reduce the incidence of MACCEs. Moreover, serial imaging of LRNC volume changes can guide therapeutic assessments and dynamic risk prediction.

While our study did not involve lipid-lowering drug interventions, existing research has shown that such medications can reduce the size of LRNC, thereby decreasing the risk of MACCEs. For example, studies have demonstrated that statins can reduce LRNC size after 1 year of continuous use. Therefore, appropriate therapeutic strategies to minimize LRNC should be considered (Brinjikji et al., 2017). This evidence is conceptually supported by mathematical modeling of plaque dynamics. Control-theoretic models, which formalize the role of inflammation in disease progression, demonstrate that statin therapy can effectively alter the growth equilibrium of atherosclerotic plaque (Formanowicz et al., 2019). These models suggest that the reduction in LRNC volume observed clinically may result from statins’ modulation of this underlying inflammatory drive, thereby shifting the system toward a more stable state.

The potential clinical value of LRNC lies in its ability to complement, rather than replace, established decision-making frameworks based on stenosis severity and symptom status. For instance, in a patient with moderate (50–69%) symptomatic stenosis or severe (≥70%) asymptomatic stenosis, where management decisions can be nuanced, the identification of a large LRNC might tip the balance toward more intensive medical therapy or invasive intervention by indicating higher plaque vulnerability (Hackam, 2021). Conversely, the absence of a significant LRNC in a stenotic plaque could reinforce a decision for conservative management. However, translating this potential into routine practice faces several challenges. Key barriers include the lack of standardized imaging and segmentation protocols across centers, the limited availability and expertise required for high-resolution vessel wall MRI, and the associated costs. Most importantly, robust prospective data are needed to define specific, clinically validated LRNC volume or proportion thresholds that provide incremental prognostic value over current standards. Future work must focus on overcoming these standardization and validation hurdles to determine if and how LRNC quantification can be systematically integrated into risk stratification models to improve patient outcomes.

A key consideration in interpreting our findings stems from the integration of different study designs. Our meta-analysis included cross-sectional and case-control studies, which assess plaque characteristics concurrent with or after an event, alongside prospective longitudinal cohorts, which assess plaques at baseline and follow for incident events. The cross-sectional and case-control analyses compellingly demonstrate a strong association between the presence and size of LRNC and MACCEs, informing our understanding of plaque vulnerability at the time of event occurrence. However, the strongest evidence for prediction—that is, using LRNC to estimate future risk in asymptomatic or stable individuals—derives primarily from the prospective cohort studies included in our cumulative incidence analyses in which LRNC was quantified by imaging before any recorded outcome event. Therefore, while our overall synthesis confirms LRNC as an important risk factor of MACCEs, clinicians and researchers should note that its prognostic utility is most directly supported by the longitudinal data, whereas its pathophysiological relevance is reinforced by the totality of evidence across all designs.

### Implications for future research

From a study design perspective, we suggest that future research should consider increasing the diversity of participants, including those from underrepresented groups such as younger patients. As overall nutritional levels improve and lifestyles change, the risk of MACCEs in younger patients should not be overlooked. Mancusi et al. (2024) have shown that carotid plaque-related indicators in younger patients can serve as risk factors for cardiovascular events. In terms of LRNC measurement, we encourage more studies to quantify the specific components of carotid plaques, including LRNC, by combining contrast-enhanced T1-weighted imaging with carotid high-resolution MRI using MR-vessel wall imaging (Takaya et al., 2006). Given that magnetic resonance-vulnerable plaque diagnostics (MR-VPD) is the only vascular plaque analysis technique certified by FDA, it is important to leverage this technology (Trivedi et al., 2004; Fan et al., 2024). Some researchers have pointed out that intraplaque hemorrhage (IPH) and fibrous cap thickness also affect the occurrence of MACCEs (Lu et al., 2019; Schindler et al., 2020). However, studies examining the synergistic effects of LRNC with other vulnerable plaque features, such as IPH and fibrous cap thickness, are limited, making systematic evaluation difficult. Therefore, we call for more research to focus on the combined data of plaque components to improve risk prediction. Additionally, the dynamic changes in LRNC size and their temporal association with MACCEs remain unclear. Although tracking the dynamic evolution of LRNC is challenging in clinical practice, we hope that future large-scale, multicenter studies will further explore these issues (Chamié & Pfau, 2024). Moreover, while it has been clinically and experimentally proven that lipid-lowering or anti-inflammatory therapies can reduce LRNC size, whether reducing LRNC can lower the risk of MACCEs still requires prospective cohort studies. Therefore, we continue to advocate for more research into the etiology of carotid plaques, particularly LRNC, to better reduce the incidence of MACCEs in the population and alleviate the medical burden on individuals and society.

## Conclusions

Our findings demonstrate that the presence and size of the lipid-rich necrotic core are consistently associated with major adverse cardiovascular and cerebrovascular events. The presence of LRNC increases MACCE risk, and strokes are more likely to occur on the same side as a lipid-core plaque. Crucially, larger LRNC volume and proportion are directly associated with event occurrence. We conclude that LRNC is a critical biomarker of plaque vulnerability, warranting its consideration in future risk stratification models. It should be noted that prospectively validated, actionable volumetric thresholds for LRNC have not yet been established. Future studies are needed to define such thresholds and to evaluate whether serial LRNC quantification can improve dynamic risk stratification in clinical trials.

##  Supplemental Information

10.7717/peerj.21214/supp-1 Supplemental Information 1 Complete search strings for each database Detailed search strategies used in PubMed, Embase, Web of Science, and Cochrane databases to identify relevant studies for the meta-analysis.

10.7717/peerj.21214/supp-2 Supplemental Information 2 Summary of effect estimates and adjusted covariates by study The effect estimates, adjustment status, and covariates adjusted for in each included study.

10.7717/peerj.21214/supp-3 Supplemental Information 3 Derivation process for missing data conversion Methods and formulas used to estimate missing statistical values (e.g., 95% CIs, standard deviations) from available data such as p-values, following Cochrane recommendations.

10.7717/peerj.21214/supp-4 Supplemental Information 4 GRADE evidence profile for key outcomes The certainty of evidence for each primary outcome assessed using the GRADE approach, including reasons for downgrading or upgrading the evidence.

10.7717/peerj.21214/supp-5 Supplemental Information 5 Characteristics of included studies Key features of the 18 studies included in the meta-analysis, including study design, participant demographics, imaging modalities, and LRNC measurement methods.

10.7717/peerj.21214/supp-6 Supplemental Information 6 Quality assessment scores of included studies The methodological quality scores of included studies using the NOS for cohort/case-control studies and the AHRQ tool for cross-sectional studies.

10.7717/peerj.21214/supp-7 Supplemental Information 7 Cumulative incidence of MACCEs in patients with LRNC Visual assessment of potential publication bias among studies included in the meta-analysis of the cumulative incidence of MACCEs in patients with LRNC.

10.7717/peerj.21214/supp-8 Supplemental Information 8 Sensitivity and subgroup analysis forest plots for ipsilateral stroke rate Forest plots from the sensitivity analysis and subgroup analyses (stratified by imaging modality, stroke type, and study design) conducted to explore and reduce heterogeneity in the analysis of ipsilateral stroke rates in patients with LRNC.

10.7717/peerj.21214/supp-9 Supplemental Information 9 Risk of bias assessment results Quality assessment scores for all included studies using the AHRQ and NOS tools, including item-level and overall ratings.

10.7717/peerj.21214/supp-10 Supplemental Information 10 Association between LRNC presence and MACCEs (HR) Potential publication bias among studies evaluating the HR for the association between the presence of LRNCs and future MACCEs.

10.7717/peerj.21214/supp-11 Supplemental Information 11 Association between LRNC presence and MACCEs (OR) Potential publication bias among studies evaluating the OR for the association between the presence of a LRNCs in carotid plaques and the occurrence of MACCEs.

10.7717/peerj.21214/supp-12 Supplemental Information 12 Ipsilateral vs. contralateral stroke occurrence Visual assessment of potential publication bias among studies comparing stroke occurrence ipsilateral versus contralateral to plaques with LRNCs. Egger’s test statistics indicate no significant asymmetry.

10.7717/peerj.21214/supp-13 Supplemental Information 13 Subgroup analysis by imaging modality (MRI vs. CT) Results of a subgroup analysis investigating the association between LRNC presence and stroke laterality, stratified by imaging modality (MRI vs. CT).

10.7717/peerj.21214/supp-14 Supplemental Information 14 Subgroup analysis by use of contrast agent in MRI studies Results of a subgroup analysis investigating the association between LRNC presence and stroke laterality, specifically within MRI studies, stratified by the use of a contrast agent.

10.7717/peerj.21214/supp-15 Supplemental Information 15 Summary of MRI sequences and plaque segmentation methods The magnetic resonance imaging sequences and plaque segmentation methodologies employed in the included studies for the identification and quantification of LRNCs.

10.7717/peerj.21214/supp-16 Supplemental Information 16 Mapping of AHA plaque classification categories to LRNC status The conventional and modified AHA histological plaque types with the presence or absence of LRNCs and their typical appearance on MRI.

10.7717/peerj.21214/supp-17 Supplemental Information 17 Meta-analysis of AHA-classified lipid-core plaques and MACCEs Results of the subgroup analysis examining the association between AHA-defined lipid-core plaques (Type IV/V) and MACCEs.

10.7717/peerj.21214/supp-18 Supplemental Information 18 Mean LRNC volume and MACCEs A visual assessment of potential publication bias among studies included in the meta-analysis of the mean LRNC volume and its correlation with MACCEs.

10.7717/peerj.21214/supp-19 Supplemental Information 19 Comparison of mean LRNC volume in ipsilateral vs. contralateral plaques in stroke patients The meta-analysis results for the difference in mean LRNC volume between ipsilateral and contralateral carotid plaques in patients with stroke.

10.7717/peerj.21214/supp-20 Supplemental Information 20 Comparison of mean LRNC volume in MACCEs vs. other ischemic symptoms The meta-analysis comparing mean LRNC volume between patients with MACCEs and those with other cerebral ischemic symptoms.

10.7717/peerj.21214/supp-21 Supplemental Information 21 Maximum LRNC volumes and MACCEs A visual assessment of potential publication bias among studies included in the meta-analysis of the maximum LRNC volumes and its correlation with MACCEs.

10.7717/peerj.21214/supp-22 Supplemental Information 22 Proportion of LRNC volume in the vessel wall and MACCEs (Compared with patients without MACCEs) A visual assessment of potential publication bias among studies included in the meta-analysis of the proportion of LRNC volume in the vessel wall and its correlation with MACCEs.

10.7717/peerj.21214/supp-23 Supplemental Information 23 Proportion of LRNC volume in the vessel wall and MACCEs (Compared with patients with cerebral ischemic symptoms) A visual assessment of potential publication bias among studies included in the meta-analysis of the proportion of LRNC volume in the vessel wall and its correlation with MACCEs.

10.7717/peerj.21214/supp-24 Supplemental Information 24 Comparison of % LRNC volume in ipsilateral vs. contralateral plaques in stroke patients The meta-analysis results for the difference in the proportion of LRNC volume between ipsilateral and contralateral plaques in stroke patients.

10.7717/peerj.21214/supp-25 Supplemental Information 25 PRISMA checklist

10.7717/peerj.21214/supp-26 Supplemental Information 26 PRISMA checklist for Abstract

10.7717/peerj.21214/supp-27 Supplemental Information 27 Target Audience

## Additional Information and Declarations

### Data Availability

The following information was supplied regarding data availability: This is a Systematic review/Meta-analysis. The code is available at Zenodo: Sun, Y. (2025). Lipid-rich necrotic core of the carotid plaque and the risk of major adverse cardiovascular and cerebrovascular events: A meta-analysis and systematic review. Zenodo. https://doi.org/10.5281/zenodo.17995743.
