# Supplementary material for: Lipid-rich necrotic core of the carotid plaque and the risk of major adverse cardiovascular and cerebrovascular events: a meta-analysis and systematic review
Source: PeerJ. 2026 May 6;14:e21214. doi: 10.7717/peerj.21214 (PMC13156956; doi:10.7717/peerj.21214)
Supplement: Supplemental Information 1 — Detailed search strategies used in PubMed, Embase, Web of Science, and Cochrane databases to identify relevant studies for the meta-analysis. [file peerj-14-21214-s001.docx]

Pubmed Search History:

(((((((((((((((((((((((((((Carotid Artery Diseases[MeSH]) OR (Artery Disease, Carotid[Title/Abstract])) OR (Artery Diseases, Carotid[Title/Abstract])) OR (Carotid Artery Disease[Title/Abstract])) OR (Arterial Diseases, Carotid[Title/Abstract])) OR (Arterial Disease, Carotid[Title/Abstract])) OR (Carotid Arterial Disease[Title/Abstract])) OR (Carotid Arterial Diseases[Title/Abstract])) OR (Carotid Artery Disorders[Title/Abstract])) OR (Artery Disorder, Carotid[Title/Abstract])) OR (Artery Disorders, Carotid[Title/Abstract])) OR (Carotid Artery Disorder[Title/Abstract])) OR (Disorders, Carotid Artery[Title/Abstract])) OR (External Carotid Artery Diseases[Title/Abstract])) OR (Arterial Diseases, External Carotid[Title/Abstract])) OR (Internal Carotid Artery Diseases[Title/Abstract])) OR (Internal Carotid Artery Disease[Title/Abstract])) OR (Carotid Atherosclerosis[Title/Abstract])) OR (Carotid Atheroscleroses[Title/Abstract])) OR (Atherosclerotic Disease, Carotid[Title/Abstract])) OR (Atherosclerotic Diseases, Carotid[Title/Abstract])) OR (Carotid Atherosclerotic Diseases[Title/Abstract])) OR (Carotid Atherosclerotic Disease[Title/Abstract])) OR (Arterial Diseases, Common Carotid[Title/Abstract])) OR (Common Carotid Artery Disease[Title/Abstract])) OR (Common Carotid Artery Diseases[Title/Abstract])) AND (((((((((((((((((((((((((((((("Ischemic Stroke"[Mesh]) OR (Ischemic Strokes[Title/Abstract])) OR (Stroke, Ischemic[Title/Abstract])) OR (Ischaemic Stroke[Title/Abstract])) OR (Ischaemic Strokes[Title/Abstract])) OR (Stroke, Ischaemic[Title/Abstract])) OR (Acute Ischemic Stroke[Title/Abstract])) OR (Acute Ischemic Strokes[Title/Abstract])) OR (Ischemic Stroke, Acute[Title/Abstract])) OR (Stroke, Acute Ischemic[Title/Abstract])) OR (Cryptogenic Ischemic Stroke[Title/Abstract])) OR (Cryptogenic Ischemic Strokes[Title/Abstract])) OR (Ischemic Stroke, Cryptogenic[Title/Abstract])) OR (Stroke, Cryptogenic Ischemic[Title/Abstract])) OR (Cryptogenic Embolism Stroke[Title/Abstract])) OR (Cryptogenic Embolism Strokes[Title/Abstract])) OR (Embolism Stroke, Cryptogenic[Title/Abstract])) OR (Stroke, Cryptogenic Embolism[Title/Abstract])) OR (Cryptogenic Stroke[Title/Abstract])) OR (Cryptogenic Strokes[Title/Abstract])) OR (Stroke, Cryptogenic[Title/Abstract])) OR (Wake-up Stroke[Title/Abstract])) OR (Stroke, Wake-up[Title/Abstract])) OR (Wake up Stroke[Title/Abstract])) OR (Wake-up Strokes[Title/Abstract])) OR (((((((((((((("Myocardial Infarction"[Mesh]) OR (Infarction, Myocardial[Title/Abstract])) OR (Infarctions, Myocardial[Title/Abstract])) OR (Myocardial Infarctions[Title/Abstract])) OR (Heart Attack[Title/Abstract])) OR (Heart Attacks[Title/Abstract])) OR (Myocardial Infarct[Title/Abstract])) OR (Infarct, Myocardial[Title/Abstract])) OR (Infarcts, Myocardial[Title/Abstract])) OR (Myocardial Infarcts[Title/Abstract])) OR (Cardiovascular Stroke[Title/Abstract])) OR (Cardiovascular Strokes[Title/Abstract])) OR (Stroke, Cardiovascular[Title/Abstract])) OR (Strokes, Cardiovascular[Title/Abstract]))) OR ((((((((((("Death, Sudden, Cardiac"[Mesh]) OR (Cardiac Sudden Death[Title/Abstract])) OR (Death, Cardiac Sudden[Title/Abstract])) OR (Sudden Death, Cardiac[Title/Abstract])) OR (Sudden Cardiac Death[Title/Abstract])) OR (Cardiac Death, Sudden[Title/Abstract])) OR (Death, Sudden Cardiac[Title/Abstract])) OR (Sudden Cardiac Arrest[Title/Abstract])) OR (Arrest, Sudden Cardiac[Title/Abstract])) OR (Cardiac Arrests, Sudden[Title/Abstract])) OR (Cardiac Arrest, Sudden[Title/Abstract]))) OR (((((((((MACCE[Title/Abstract]) OR (Major Adverse Cardiovascular[Title/Abstract] AND Cerebrovascular Events[Title/Abstract])) OR (Main Adverse Cardiovascular[Title/Abstract] AND Cerebrovascular Events[Title/Abstract])) OR (Major Adverse Cardiac[Title/Abstract] AND Cerebrovascular Events[Title/Abstract])) OR (Main Adverse Cardiac[Title/Abstract] AND Cerebrovascular Events[Title/Abstract])) OR (Major Adverse Cardiovascular[Title/Abstract] AND Cerebrovascular Event[Title/Abstract])) OR (Main Adverse Cardiovascular[Title/Abstract] AND Cerebrovascular Event[Title/Abstract])) OR (Major Adverse Cardiac[Title/Abstract] AND Cerebrovascular Event[Title/Abstract])) OR (Main Adverse Cardiac[Title/Abstract] AND Cerebrovascular Event[Title/Abstract]))) OR ((((((((((("Cardiovascular Diseases"[Mesh]) OR (Cardiovascular Disease[Title/Abstract])) OR (Disease, Cardiovascular[Title/Abstract])) OR (Cardiac Events[Title/Abstract])) OR (Cardiac Event[Title/Abstract])) OR (Event, Cardiac[Title/Abstract])) OR (Adverse Cardiac Event[Title/Abstract])) OR (Adverse Cardiac Events[Title/Abstract])) OR (Cardiac Event, Adverse[Title/Abstract])) OR (Cardiac Events, Adverse[Title/Abstract])) OR (Major Adverse Cardiac Events[Title/Abstract]))) OR (((((((((((((((((((((((((("Cerebrovascular Disorders"[Mesh]) OR (Cerebrovascular Disorder[Title/Abstract])) OR (Brain Vascular Disorders[Title/Abstract])) OR (Brain Vascular Disorder[Title/Abstract])) OR (Vascular Disorder, Brain[Title/Abstract])) OR (Vascular Disorders, Brain[Title/Abstract])) OR (Intracranial Vascular Disorders[Title/Abstract])) OR (Intracranial Vascular Disorder[Title/Abstract])) OR (Vascular Disorder, Intracranial[Title/Abstract])) OR (Vascular Disorders, Intracranial[Title/Abstract])) OR (Vascular Diseases, Intracranial[Title/Abstract])) OR (Intracranial Vascular Disease[Title/Abstract])) OR (Intracranial Vascular Diseases[Title/Abstract])) OR (Vascular Disease, Intracranial[Title/Abstract])) OR (Cerebrovascular Diseases[Title/Abstract])) OR (Cerebrovascular Disease[Title/Abstract])) OR (Disease, Cerebrovascular[Title/Abstract])) OR (Diseases, Cerebrovascular[Title/Abstract])) OR (Cerebrovascular Insufficiency[Title/Abstract])) OR (Cerebrovascular Insufficiencies[Title/Abstract])) OR (Insufficiencies, Cerebrovascular[Title/Abstract])) OR (Insufficiency, Cerebrovascular[Title/Abstract])) OR (Cerebrovascular Occlusion[Title/Abstract])) OR (Cerebrovascular Occlusions[Title/Abstract])) OR (Occlusion, Cerebrovascular[Title/Abstract])) OR (Occlusions, Cerebrovascular[Title/Abstract])))) AND ((((((Lipid Droplets[Title/Abstract]) OR (lipid-rich necrotic core[Title/Abstract])) OR (necrotic core[Title/Abstract])) OR (LCBI[Title/Abstract])) OR (lipid core[Title/Abstract])) OR (lipid-core burden index[Title/Abstract]))

Web of Science search history:

1: (((((((TS=( (Cardiovascular Disease) OR (Cardiac Events) OR (Cardiac Event) OR (Adverse Cardiac Event) OR (Adverse Cardiac Events) OR (Major Adverse Cardiac Events) )) OR TS=( (Cerebrovascular Disorder) OR (Brain Vascular Disorders) OR (Brain Vascular Disorder) OR (Intracranial Vascular Disorders) OR (Intracranial Vascular Disorder) OR (Intracranial Vascular Disease) OR (Intracranial Vascular Diseases) OR (Cerebrovascular Diseases) OR (Cerebrovascular Disease) OR (Cerebrovascular Insufficiency) OR (Cerebrovascular Insufficiencies) OR (Cerebrovascular Occlusion) OR (Cerebrovascular Occlusions) )) OR TS=((MACCE) OR (Major Adverse Cardiovascular and Cerebrovascular Events) OR (Main Adverse Cardiovascular and Cerebrovascular Events) OR (Major Adverse Cardiac and Cerebrovascular Events) OR (Main Adverse Cardiac and Cerebrovascular Events) OR (Major Adverse Cardiovascular and Cerebrovascular Event) OR (Main Adverse Cardiovascular and Cerebrovascular Event) OR (Major Adverse Cardiac and Cerebrovascular Event) OR (Main Adverse Cardiac and Cerebrovascular Event) )) OR TS=( (Cardiac Sudden Death) OR (Sudden Cardiac Death) OR (Sudden Cardiac Arrest) )) OR TS=( (Myocardial Infarctions) OR (Heart Attack) OR (Heart Attacks) OR (Myocardial Infarct) OR (Myocardial Infarcts) OR (Cardiovascular Stroke) OR (Cardiovascular Strokes) )) OR TS=( (Ischemic Strokes) OR (Ischaemic Stroke) OR (Ischaemic Strokes) OR (Acute Ischemic Stroke) OR (Acute Ischemic Strokes) OR (Cryptogenic Ischemic Stroke) OR (Cryptogenic Ischemic Strokes) OR (Cryptogenic Embolism Stroke) OR (Cryptogenic Embolism Strokes) OR (Cryptogenic Stroke) OR (Cryptogenic Strokes) OR (Wake-up Stroke) OR (Wake up Stroke) OR (Wake-up Strokes) )) ) and Preprint Citation Index (Exclude – Database)

2: TS=((Lipid Droplets) OR (lipid-rich necrotic core) OR (necrotic core) OR (LCBI) OR (lipid core) OR (lipid-core burden index) ) and Preprint Citation Index (Exclude – Database)

3: ((((((((((((((TS=(Carotid Artery Diseases)) OR TS=(Carotid Artery Disease)) OR TS=(Carotid Arterial Disease)) OR TS=(Carotid Arterial Diseases)) OR TS=(Carotid Artery Disorders)) OR TS=(Carotid Artery Disorder)) OR TS=(External Carotid Artery Diseases)) OR TS=(Internal Carotid Artery Diseases)) OR TS=(Internal Carotid Artery Disease)) OR TS=(Carotid Atherosclerosis)) OR TS=(Carotid Atheroscleroses)) OR TS=(Carotid Atherosclerotic Diseases)) OR TS=(Carotid Atherosclerotic Disease)) OR TS=(Common Carotid Artery Disease)) OR TS=(Common Carotid Artery Diseases) and Preprint Citation Index (Exclude – Database)

4: #3 AND #2 AND #1 and Preprint Citation Index (Exclude – Database)

Embase search history:

#11 #8 AND #9 AND #10

#10 ('carotid artery diseases'/exp OR 'carotid artery diseases' OR 'artery disease, carotid' OR 'artery diseases, carotid' OR 'carotid artery disease'/exp OR 'carotid artery disease' OR 'arterial diseases, carotid' OR 'arterial disease, carotid' OR 'carotid arterial disease'/exp OR 'carotid arterial disease' OR 'carotid arterial diseases' OR 'carotid artery disorders' OR 'artery disorder, carotid' OR 'artery disorders, carotid' OR 'carotid artery disorder'/exp OR 'carotid artery disorder' OR 'disorders, carotid artery' OR 'external carotid artery diseases' OR 'arterial diseases, external carotid' OR 'internal carotid artery diseases' OR 'arterial diseases, internal carotid' OR 'internal carotid artery disease' OR 'carotid atherosclerosis'/exp OR 'carotid atherosclerosis' OR 'carotid atheroscleroses' OR 'atherosclerotic disease, carotid' OR 'atherosclerotic diseases, carotid' OR 'carotid atherosclerotic diseases' OR 'carotid atherosclerotic disease'/exp OR 'carotid atherosclerotic disease' OR 'arterial diseases, common carotid' OR 'common carotid artery disease' OR 'common carotid artery diseases') AND [1967-2024]/py

#9 ('lipid droplets'/exp OR 'lipid droplets' OR 'lipid-rich necrotic core' OR 'necrotic core'/exp OR 'necrotic core' OR 'lcbi' OR 'lipid core' OR 'lipid-core burden index') AND [1967-2024]/py

#8 #1 OR #2 OR #3 OR #4 OR #5 OR #6

#6 ('ischemic stroke'/exp OR 'ischemic stroke' OR 'ischemic strokes' OR 'stroke, ischemic' OR 'ischaemic stroke'/exp OR 'ischaemic stroke' OR 'ischaemic strokes' OR 'stroke, ischaemic' OR 'acute ischemic stroke'/exp OR 'acute ischemic stroke' OR 'acute ischemic strokes' OR 'ischemic stroke, acute' OR 'stroke, acute ischemic' OR 'cryptogenic ischemic stroke'/exp OR 'cryptogenic ischemic stroke' OR 'cryptogenic ischemic strokes' OR 'ischemic stroke, cryptogenic' OR 'stroke, cryptogenic ischemic' OR 'cryptogenic embolism stroke' OR 'cryptogenic embolism strokes' OR 'embolism stroke, cryptogenic' OR 'stroke, cryptogenic embolism' OR 'cryptogenic stroke'/exp OR 'cryptogenic stroke' OR 'cryptogenic strokes' OR 'stroke, cryptogenic' OR 'wake-up stroke'/exp OR 'wake-up stroke' OR 'stroke, wake-up' OR 'wake up stroke'/exp OR 'wake up stroke' OR 'wake-up strokes') AND [1967-2024]/py

#5 ('myocardial infarction'/exp OR 'myocardial infarction' OR 'infarction, myocardial' OR 'infarctions, myocardial' OR 'myocardial infarctions' OR 'heart attack'/exp OR 'heart attack' OR 'heart attacks' OR 'myocardial infarct'/exp OR 'myocardial infarct' OR 'infarct, myocardial' OR 'infarcts, myocardial' OR 'myocardial infarcts' OR 'cardiovascular stroke' OR 'cardiovascular strokes' OR 'stroke, cardiovascular' OR 'strokes, cardiovascular') AND [1967-2024]/py

#4 ('cardiovascular diseases'/exp OR 'cardiovascular diseases' OR 'cardiovascular disease'/exp OR 'cardiovascular disease' OR 'disease, cardiovascular'/exp OR 'disease, cardiovascular' OR 'cardiac events' OR 'cardiac event' OR 'event, cardiac' OR 'adverse cardiac event' OR 'adverse cardiac events' OR 'cardiac event, adverse' OR 'cardiac events, adverse' OR 'major adverse cardiac events'/exp OR 'major adverse cardiac events') AND [1967-2024]/py

#3 ('cerebrovascular disorders'/exp OR 'cerebrovascular disorders' OR 'cerebrovascular disorder'/exp OR 'cerebrovascular disorder' OR 'brain vascular disorders' OR 'brain vascular disorder' OR 'vascular disorder, brain' OR 'vascular disorders, brain' OR 'intracranial vascular disorders' OR 'intracranial vascular disorder' OR 'vascular disorder, intracranial' OR 'vascular disorders, intracranial' OR 'vascular diseases, intracranial' OR 'intracranial vascular disease' OR 'intracranial vascular diseases' OR 'vascular disease, intracranial' OR 'cerebrovascular diseases' OR 'cerebrovascular disease'/exp OR 'cerebrovascular disease' OR 'disease, cerebrovascular' OR 'diseases, cerebrovascular' OR 'cerebrovascular insufficiency'/exp OR 'cerebrovascular insufficiency' OR 'cerebrovascular insufficiencies' OR 'insufficiencies, cerebrovascular' OR 'insufficiency, cerebrovascular' OR 'cerebrovascular occlusion'/exp OR 'cerebrovascular occlusion' OR 'cerebrovascular occlusions' OR 'occlusion, cerebrovascular' OR 'occlusions, cerebrovascular') AND [1967-2024]/py

#2 ('macce' OR 'major adverse cardiovascular and cerebrovascular events' OR 'main adverse cardiovascular and cerebrovascular events' OR 'major adverse cardiac and cerebrovascular events' OR 'main adverse cardiac and cerebrovascular events' OR 'major adverse cardiovascular and cerebrovascular event'/exp OR 'major adverse cardiovascular and cerebrovascular event' OR 'main adverse cardiovascular and cerebrovascular event' OR 'major adverse cardiac and cerebrovascular event'/exp OR 'major adverse cardiac and cerebrovascular event' OR 'main adverse cardiac and cerebrovascular event') AND [1967-2024]/py

#1 ('death, sudden, cardiac'/exp OR 'death, sudden, cardiac' OR 'cardiac sudden death'/exp OR 'cardiac sudden death' OR 'death, cardiac sudden' OR 'sudden death, cardiac' OR 'sudden cardiac death'/exp OR 'sudden cardiac death' OR 'cardiac death, sudden'/exp OR 'cardiac death, sudden' OR 'death, sudden cardiac'/exp OR 'death, sudden cardiac' OR 'sudden cardiac arrest'/exp OR 'sudden cardiac arrest' OR 'arrest, sudden cardiac' OR 'cardiac arrests, sudden' OR 'cardiac arrest, sudden') AND [1967-2024]/py

Cochrane search history:

#1 MeSH descriptor: [Cardiovascular Diseases] explode all trees

#2 (Cardiovascular Disease):ti,ab,kw OR (Disease, Cardiovascular):ti,ab,kw OR (Cardiac Events):ti,ab,kw OR (Cardiac Event):ti,ab,kw OR (Event, Cardiac):ti,ab,kw OR (Adverse Cardiac Event):ti,ab,kw OR (Adverse Cardiac Events):ti,ab,kw OR (Cardiac Event, Adverse):ti,ab,kw OR (Cardiac Events, Adverse):ti,ab,kw OR (Major Adverse Cardiac Events):ti,ab,kw

#3 #1 OR #2

#4 MeSH descriptor: [Cerebrovascular Disorders] explode all trees

#5 (Cerebrovascular Disorder):ti,ab,kw OR (Brain Vascular Disorders):ti,ab,kw OR (Brain Vascular Disorder):ti,ab,kw OR (Vascular Disorder, Brain):ti,ab,kw OR (Vascular Disorders, Brain):ti,ab,kw OR (Intracranial Vascular Disorders):ti,ab,kw OR (Intracranial Vascular Disorder):ti,ab,kw OR (Vascular Disorder, Intracranial):ti,ab,kw OR (Vascular Disorders, Intracranial):ti,ab,kw OR (Vascular Diseases, Intracranial):ti,ab,kw OR (Intracranial Vascular Disease):ti,ab,kw OR (Intracranial Vascular Diseases):ti,ab,kw OR (Vascular Disease, Intracranial):ti,ab,kw OR (Cerebrovascular Diseases):ti,ab,kw OR (Cerebrovascular Disease):ti,ab,kw OR (Disease, Cerebrovascular):ti,ab,kw OR (Diseases, Cerebrovascular):ti,ab,kw OR (Cerebrovascular Insufficiency):ti,ab,kw OR (Cerebrovascular Insufficiencies):ti,ab,kw OR (Insufficiencies, Cerebrovascular):ti,ab,kw OR (Insufficiency, Cerebrovascular):ti,ab,kw OR (Cerebrovascular Occlusion):ti,ab,kw OR (Cerebrovascular Occlusions):ti,ab,kw OR (Occlusion, Cerebrovascular):ti,ab,kw OR (Occlusions, Cerebrovascular):ti,ab,kw

#6 #4 OR #5

#7 (MACCE):ti,ab,kw OR (Major Adverse Cardiovascular and Cerebrovascular Events):ti,ab,kw OR (Main Adverse Cardiovascular and Cerebrovascular Events):ti,ab,kw OR (Major Adverse Cardiac and Cerebrovascular Events):ti,ab,kw OR (Main Adverse Cardiac and Cerebrovascular Events):ti,ab,kw OR (Major Adverse Cardiovascular and Cerebrovascular Event):ti,ab,kw OR (Main Adverse Cardiovascular and Cerebrovascular Event):ti,ab,kw OR (Major Adverse Cardiac and Cerebrovascular Event):ti,ab,kw OR (Main Adverse Cardiac and Cerebrovascular Event):ti,ab,kw

#8 MeSH descriptor: [Death, Sudden, Cardiac] explode all trees

#9 (Cardiac Sudden Death):ti,ab,kw OR (Death, Cardiac Sudden):ti,ab,kw OR (Sudden Death, Cardiac):ti,ab,kw OR (Sudden Cardiac Death):ti,ab,kw OR (Cardiac Death, Sudden):ti,ab,kw OR (Death, Sudden Cardiac):ti,ab,kw OR (Sudden Cardiac Arrest):ti,ab,kw OR (Arrest, Sudden Cardiac):ti,ab,kw OR (Cardiac Arrests, Sudden):ti,ab,kw OR (Cardiac Arrest, Sudden):ti,ab,kw

#10 #8 OR #9

#11 MeSH descriptor: [Myocardial Infarction] explode all trees

#12 (Infarction, Myocardial):ti,ab,kw OR (Infarctions, Myocardial):ti,ab,kw OR (Myocardial Infarctions):ti,ab,kw OR (Heart Attack):ti,ab,kw OR (Heart Attacks):ti,ab,kw OR (Myocardial Infarct):ti,ab,kw OR (Infarct, Myocardial):ti,ab,kw OR (Infarcts, Myocardial):ti,ab,kw OR (Myocardial Infarcts):ti,ab,kw OR (Cardiovascular Stroke):ti,ab,kw OR (Cardiovascular Strokes):ti,ab,kw OR (Stroke, Cardiovascular):ti,ab,kw OR (Strokes, Cardiovascular):ti,ab,kw

#13 #11 OR #12

#14 MeSH descriptor: [Ischemic Stroke] explode all trees

#15 (Ischemic Strokes):ti,ab,kw OR (Stroke, Ischemic):ti,ab,kw OR (Ischaemic Stroke):ti,ab,kw OR (Ischaemic Strokes):ti,ab,kw OR (Stroke, Ischaemic):ti,ab,kw OR (Acute Ischemic Stroke):ti,ab,kw OR (Acute Ischemic Strokes):ti,ab,kw OR (Ischemic Stroke, Acute):ti,ab,kw OR (Stroke, Acute Ischemic):ti,ab,kw OR (Cryptogenic Ischemic Stroke):ti,ab,kw OR (Cryptogenic Ischemic Strokes):ti,ab,kw OR (Ischemic Stroke, Cryptogenic):ti,ab,kw OR (Stroke, Cryptogenic Ischemic):ti,ab,kw OR (Cryptogenic Embolism Stroke):ti,ab,kw OR (Cryptogenic Embolism Strokes):ti,ab,kw OR (Embolism Stroke, Cryptogenic):ti,ab,kw OR (Stroke, Cryptogenic Embolism):ti,ab,kw OR (Cryptogenic Stroke):ti,ab,kw OR (Cryptogenic Strokes):ti,ab,kw OR (Stroke, Cryptogenic):ti,ab,kw OR (Wake-up Stroke):ti,ab,kw OR (Stroke, Wake-up):ti,ab,kw OR (Wake up Stroke):ti,ab,kw OR (Wake-up Strokes):ti,ab,kw

#16 #15 OR #14

#17 #3 OR #6 OR #7 OR #10 OR #13 OR #16

#18 (Lipid Droplets):ti,ab,kw OR (lipid-rich necrotic core):ti,ab,kw OR (necrotic core):ti,ab,kw OR (LCBI):ti,ab,kw OR (lipid core):ti,ab,kw OR (lipid-core burden index):ti,ab,kw

#19 #17 AND #18

#20 (Carotid Artery Diseases):ti,ab,kw OR (Artery Disease, Carotid):ti,ab,kw OR (Artery Diseases, Carotid):ti,ab,kw OR (Carotid Artery Disease):ti,ab,kw OR (Arterial Diseases, Carotid):ti,ab,kw OR (Arterial Disease, Carotid):ti,ab,kw OR (Carotid Arterial Disease):ti,ab,kw OR (Carotid Arterial Diseases):ti,ab,kw OR (Carotid Artery Disorders):ti,ab,kw OR (Artery Disorder, Carotid):ti,ab,kw OR (Artery Disorders, Carotid):ti,ab,kw OR (Carotid Artery Disorder):ti,ab,kw OR (Disorders, Carotid Artery):ti,ab,kw OR (External Carotid Artery Diseases):ti,ab,kw OR (Arterial Diseases, External Carotid):ti,ab,kw OR (Internal Carotid Artery Diseases):ti,ab,kw OR (Internal Carotid Artery Disease):ti,ab,kw OR (Carotid Atherosclerosis):ti,ab,kw OR (Carotid Atheroscleroses):ti,ab,kw OR (Atherosclerotic Disease, Carotid):ti,ab,kw OR (Atherosclerotic Diseases, Carotid):ti,ab,kw OR (Carotid Atherosclerotic Diseases):ti,ab,kw OR (Carotid Atherosclerotic Disease):ti,ab,kw OR (Arterial Diseases, Common Carotid):ti,ab,kw OR (Common Carotid Artery Disease):ti,ab,kw OR (Common Carotid Artery Diseases):ti,ab,kw

#21 #19 AND #20
