# Supplementary material for: Lipid-rich necrotic core of the carotid plaque and the risk of major adverse cardiovascular and cerebrovascular events: a meta-analysis and systematic review
Source: PeerJ. 2026 May 6;14:e21214. doi: 10.7717/peerj.21214 (PMC13156956; doi:10.7717/peerj.21214)
Supplement: Supplemental Information 2 — The effect estimates, adjustment status, and covariates adjusted for in each included study. [file peerj-14-21214-s002.docx]

Summary of effect estimates and adjusted covariates by study.

| NO. | Author | Year | Effect Estimate | Adjusted? (Y/N) | Adjusted Covariates (Summary) |
| --- | --- | --- | --- | --- | --- |
| 1 | TangYX | 2023 | OR | N | No adjustment for covariates. |
| 2 | DalagerS | 2007 | OR | N | No adjustment for covariates. |
| 3 | BrunnerG | 2021 | HR | Y | Age, sex, race, study center, smoking, BMI, blood glucose, diabetes, SBP, DBP, HDL-C, non-HDL-C, use of BP/cholesterol/aspirin/diabetes meds, hs-CRP. (+ max wall thickness for plaque components). |
| 5 | ToornJ | 2022 | HR | Y | Base model: age (spline), corrected total cholesterol, HDL-C, corrected SBP, current smoking, diabetes. |
| 6 | XuYL | 2016 | OR | Y | Age, gender, BMI, hypertension, smoking, diabetes, hyperlipidemia. |
| 7 | LuMM | 2022 | OR | Y | Age, sex, BMI, diabetes mellitus, maximum NWI. |
| 8 | GrimmJ | 2013 | OR | N | No adjustment for covariates. |
| 9 | SaamT | 2016 | OR | N | No adjustment for covariates. |
| 11 | GuoDL | 2022 | OR | N | No adjustment for covariates. |
| 12 | HyafilF | 2016 | OR | N | No adjustment for covariates. |
