# Supplementary material for: Lipid-rich necrotic core of the carotid plaque and the risk of major adverse cardiovascular and cerebrovascular events: a meta-analysis and systematic review
Source: PeerJ. 2026 May 6;14:e21214. doi: 10.7717/peerj.21214 (PMC13156956; doi:10.7717/peerj.21214)
Supplement: Supplemental Information 4 — The certainty of evidence for each primary outcome assessed using the GRADE approach, including reasons for downgrading or upgrading the evidence. [file peerj-14-21214-s004.docx]

| **№ of studies** | **Certainty assessment** | | | | | | **Effect** | | | **Certainty** | **Importance** |
| --- | --- | --- | --- | --- | --- | --- | --- | --- | --- | --- | --- |
|  | **Study design** | **Risk of bias** | **Inconsistency** | **Indirectness** | **Imprecision** | **Other considerations** | **№ of events** | **№ of individuals** | **Rate (95% CI)** |  |  |
| the cumulative incidence of MACCEs in patients with LRNC of carotid plaques (follow-up: mean 31.2 years) | | | | | | | | | | | |
| 2 | non-randomised studies | not serious | serious^a^ | not serious | not serious | none | 3 | 723 | event rate 102 per 1000 (0.03 to 0.295) | ⨁⨁⨁◯ Moderate^a^ | CRITICAL |
| stroke proportion in patients with LRNC of carotid plaques ipsilateral or contralateral | | | | | | | | | | | |
| 5 | non-randomised studies | not serious | not serious | not serious | not serious | none | 1 | 157 | event rate 611 per 1000 (533 to 0.684) | ⨁⨁⨁⨁ High | CRITICAL |
| the correlation between mean LRNC volume and MACCEs. | | | | | | | | | | | |
| 3 | non-randomised studies | not serious | not serious | not serious | not serious | none | - | 481 | MD 8.284 (1.353 to 15.215) | ⨁⨁⨁⨁ High | IMPORTANT |
| the correlation between max LRNC area and stroke | | | | | | | | | | | |
| 2 | non-randomised studies | not serious | not serious | not serious | not serious | none | - | 56 | MD 8.68 (4.095 to 13.265) | ⨁⨁⨁⨁ High | IMPORTANT |
| the correlation between % LRNC volume and MACCEs | | | | | | | | | | | |
| 3 | non-randomised studies | not serious | not serious | not serious | serious^b^ | none | - | 481 | MD 0.471 % (0.073 to 0.868) | ⨁⨁⨁◯ Moderate^b^ | IMPORTANT |

Explanations

a. follow-up periods differ

b. different methods
