# Supplementary material for: Lipid-rich necrotic core of the carotid plaque and the risk of major adverse cardiovascular and cerebrovascular events: a meta-analysis and systematic review
Source: PeerJ. 2026 May 6;14:e21214. doi: 10.7717/peerj.21214 (PMC13156956; doi:10.7717/peerj.21214)
Supplement: Supplemental Information 6 — The methodological quality scores of included studies using the NOS for cohort/case-control studies and the AHRQ tool for cross-sectional studies. [file peerj-14-21214-s006.docx]

Quality assessment scores of included studies.

| NO. | ID | Year | Type | 1 | 2 | 3 | 4 | 5 | 6 | 7 | 8 | 9 | 10 | 11 | score | overall |
| --- | --- | --- | --- | --- | --- | --- | --- | --- | --- | --- | --- | --- | --- | --- | --- | --- |
| NOS |  |  |  |  |  |  |  |  |  |  |  |  |  |  |  |  |
| 1 | TangYX | 2023 | case control study | 1 | 0 | 0 | 0 | 1 | 0 | 1 | 1 | 0 |  |  | 4 | medium |
| 3 | BrunnerG | 2021 | cohort study | 1 | 1 | 1 | 1 | 0 | 0 | 1 | 1 | 1 |  |  | 7 | high |
| 4 | BosD | 2021 | cohort study | 1 | 1 | 1 | 1 | 0 | 0 | 1 | 1 | 1 |  |  | 7 | high |
| 5 | ToornJ | 2022 | cohort study | 1 | 1 | 1 | 1 | 0 | 0 | 1 | 1 | 1 |  |  | 7 | high |
| 9 | SaamT | 2016 | cohort study | 1 | 1 | 1 | 1 | 0 | 0 | 1 | 0 | 0 |  |  | 5 | medium |
| 10 | CheFL | 2021 | cohort study | 1 | 1 | 1 | 0 | 0 | 1 | 1 | 1 | 1 |  |  | 7 | high |
| 13 | SongJ | 2021 | cohort study | 1 | 1 | 1 | 0 | 0 | 1 | 1 | 0 | 0 |  |  | 5 | medium |
| 15 | MaggeR | 2013 | cohort study | 1 | 1 | 1 | 0 | 0 | 1 | 1 | 0 | 0 |  |  | 5 | medium |
| AHRQ |  |  |  |  |  |  |  |  |  |  |  |  |  |  |  |  |
| 2 | DalagerS | 2007 | cross-sectional study | 0 | 0 | 0 | 1 | 0 | 0 | 1 | 0 | 0 | 0 | 0 | 2 | low |
| 6 | XuYL | 2016 | cross-sectional study | 0 | 1 | 1 | 0 | 0 | 0 | 0 | 0 | 0 | 0 | 0 | 2 | low |
| 7 | LuMM | 2022 | cross-sectional study | 0 | 0 | 1 | 0 | 0 | 0 | 0 | 0 | 1 | 1 | 0 | 3 | low |
| 8 | GrimmJ | 2013 | cross-sectional study | 0 | 0 | 1 | 0 | 0 | 0 | 0 | 0 | 1 | 1 | 0 | 3 | medium |
| 11 | GuoDL | 2022 | cross-sectional study | 0 | 0 | 0 | 0 | 0 | 0 | 1 | 0 | 1 | 1 | 0 | 3 | medium |
| 12 | HyafilF | 2016 | cross-sectional study | 0 | 0 | 0 | 0 | 0 | 0 | 1 | 0 | 1 | 1 | 0 | 3 | medium |
| 15 | GiannottiN | 2021 | cross-sectional study | 1 | 1 | 1 | 1 | 1 | 0 | 0 | 0 | 1 | 1 | 1 | 8 | high |
