# Supplementary figures and images for: Lipid-rich necrotic core of the carotid plaque and the risk of major adverse cardiovascular and cerebrovascular events: a meta-analysis and systematic review
Source: PeerJ. 2026 May 6;14:e21214. doi: 10.7717/peerj.21214 (PMC13156956; doi:10.7717/peerj.21214)

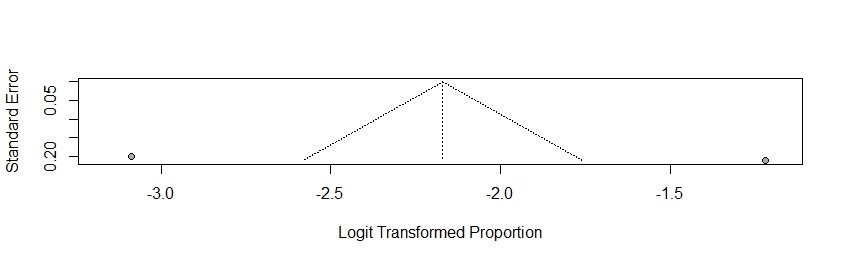

Supplement: Supplemental Information 7 — Visual assessment of potential publication bias among studies included in the meta-analysis of the cumulative incidence of MACCEs in patients with LRNC. [file peerj-14-21214-s007.jpg]

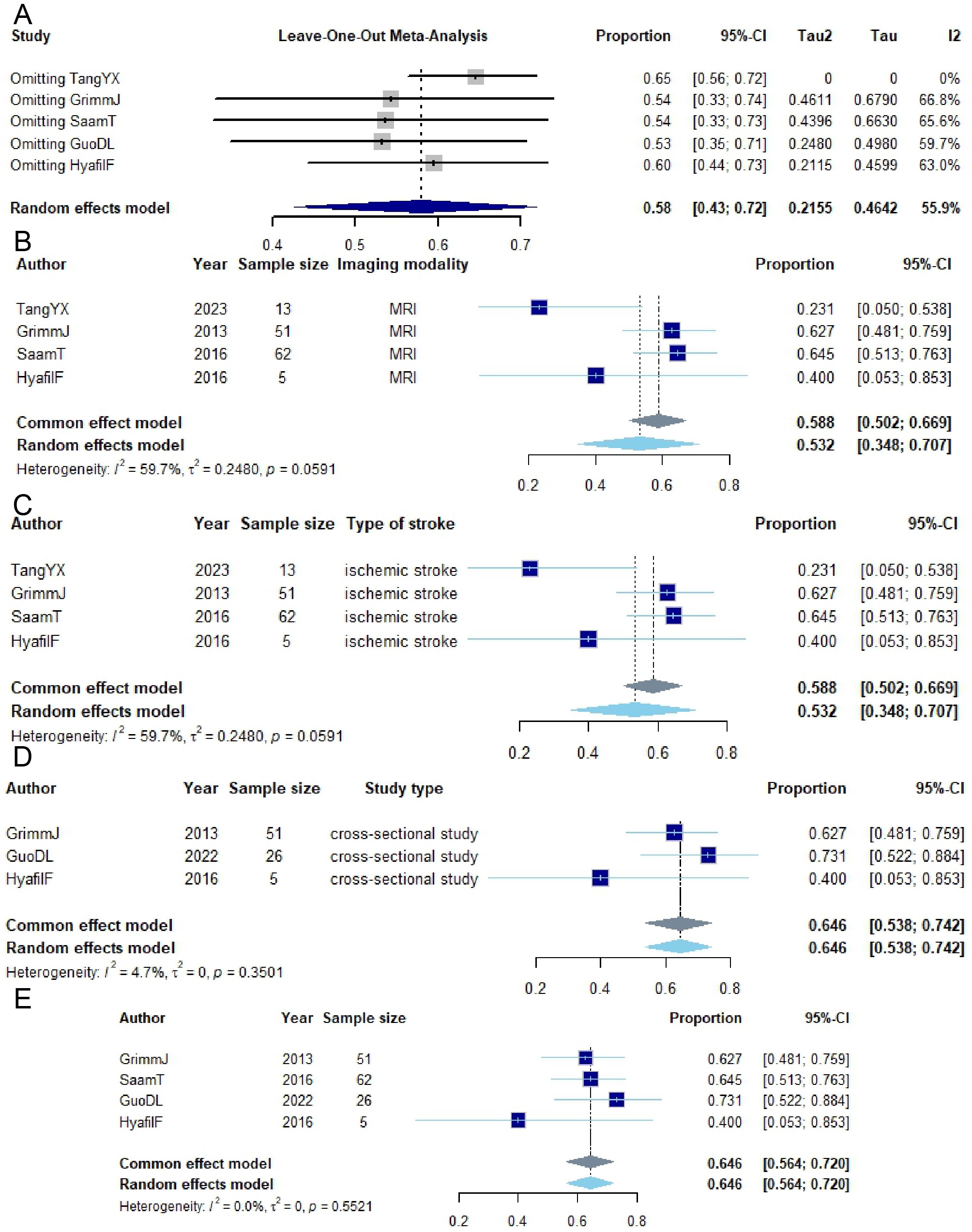

Supplement: Supplemental Information 8 — Forest plots from the sensitivity analysis and subgroup analyses (stratified by imaging modality, stroke type, and study design) conducted to explore and reduce heterogeneity in the analysis of ipsilateral stroke rates in patients with LRNC. [file peerj-14-21214-s008.jpg]

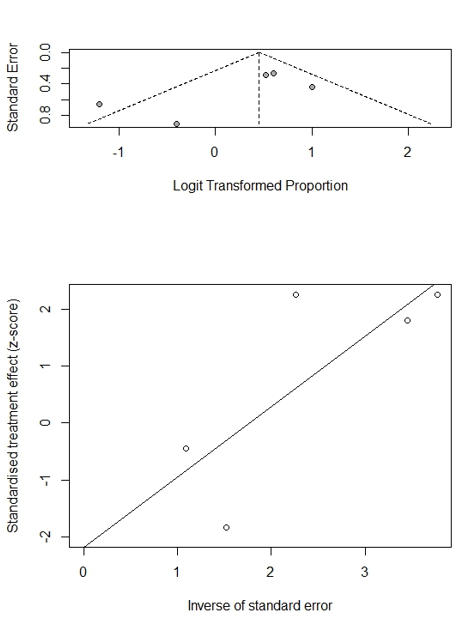

Supplement: Supplemental Information 9 — Quality assessment scores for all included studies using the AHRQ and NOS tools, including item-level and overall ratings. [file peerj-14-21214-s009.jpg]

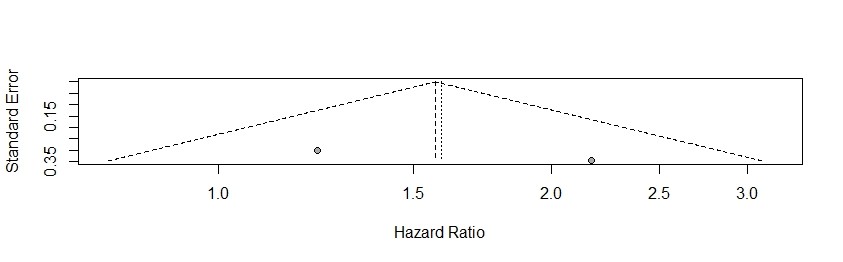

Supplement: Supplemental Information 10 — Potential publication bias among studies evaluating the HR for the association between the presence of LRNCs and future MACCEs. [file peerj-14-21214-s010.jpg]

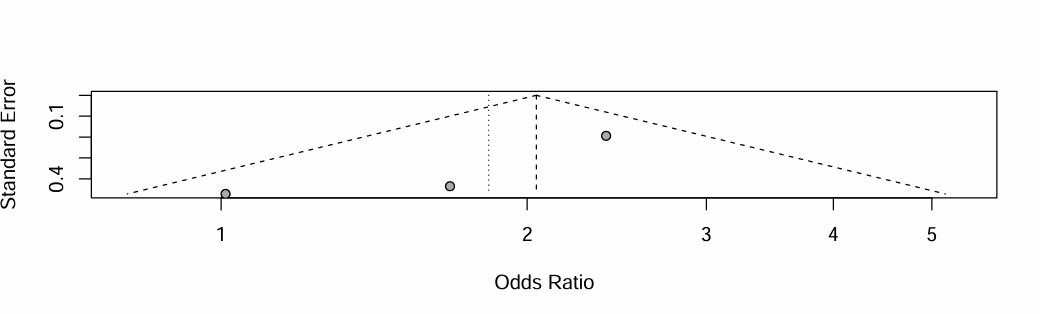

Supplement: Supplemental Information 11 — Potential publication bias among studies evaluating the OR for the association between the presence of a LRNCs in carotid plaques and the occurrence of MACCEs. [file peerj-14-21214-s011.jpg]

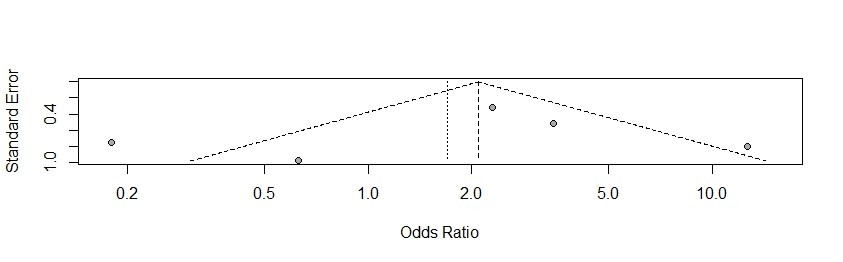

Supplement: Supplemental Information 12 — Visual assessment of potential publication bias among studies comparing stroke occurrence ipsilateral versus contralateral to plaques with LRNCs. Egger’s test statistics indicate no significant asymmetry. [file peerj-14-21214-s012.jpg]

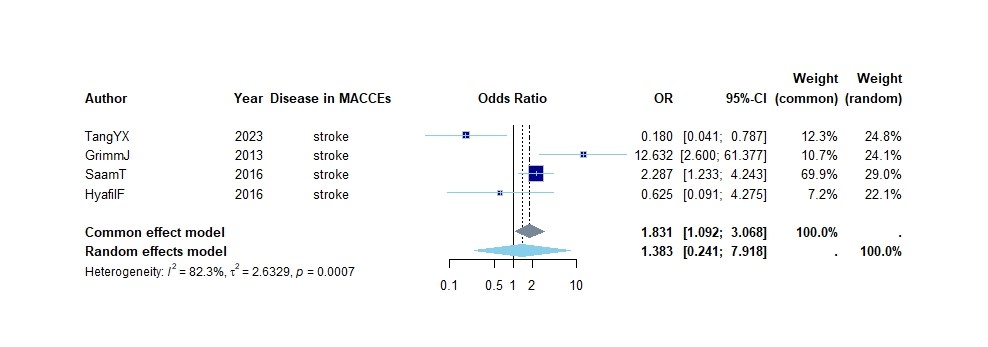

Supplement: Supplemental Information 13 — Results of a subgroup analysis investigating the association between LRNC presence and stroke laterality, stratified by imaging modality (MRI vs. CT). [file peerj-14-21214-s013.jpg]

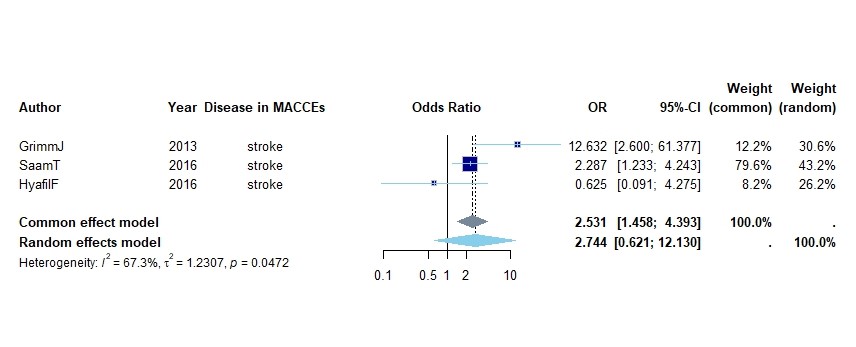

Supplement: Supplemental Information 14 — Results of a subgroup analysis investigating the association between LRNC presence and stroke laterality, specifically within MRI studies, stratified by the use of a contrast agent. [file peerj-14-21214-s014.jpg]

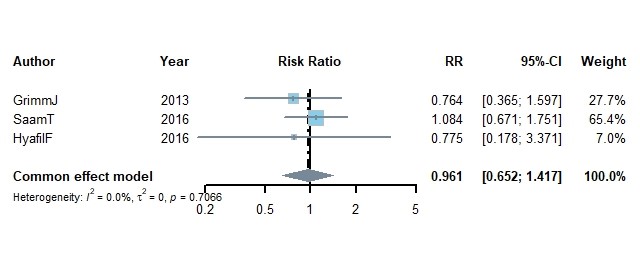

Supplement: Supplemental Information 17 — Results of the subgroup analysis examining the association between AHA-defined lipid-core plaques (Type IV/V) and MACCEs. [file peerj-14-21214-s017.jpg]

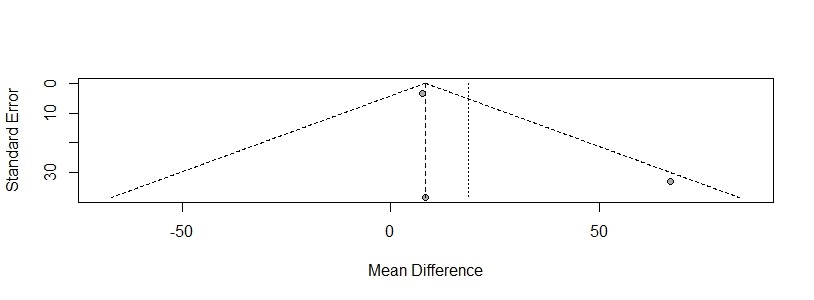

Supplement: Supplemental Information 18 — A visual assessment of potential publication bias among studies included in the meta-analysis of the mean LRNC volume and its correlation with MACCEs. [file peerj-14-21214-s018.jpg]

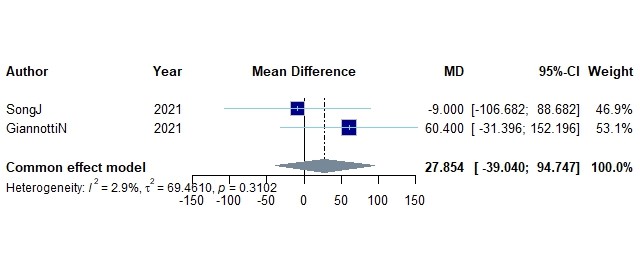

Supplement: Supplemental Information 19 — The meta-analysis results for the difference in mean LRNC volume between ipsilateral and contralateral carotid plaques in patients with stroke. [file peerj-14-21214-s019.jpg]

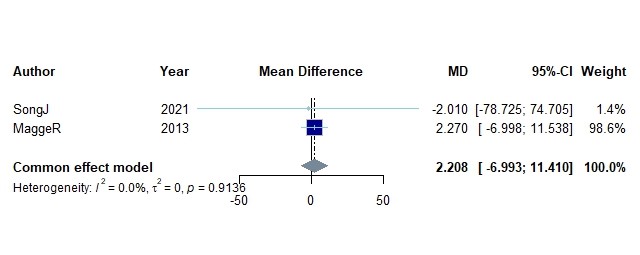

Supplement: Supplemental Information 20 — The meta-analysis comparing mean LRNC volume between patients with MACCEs and those with other cerebral ischemic symptoms. [file peerj-14-21214-s020.jpg]

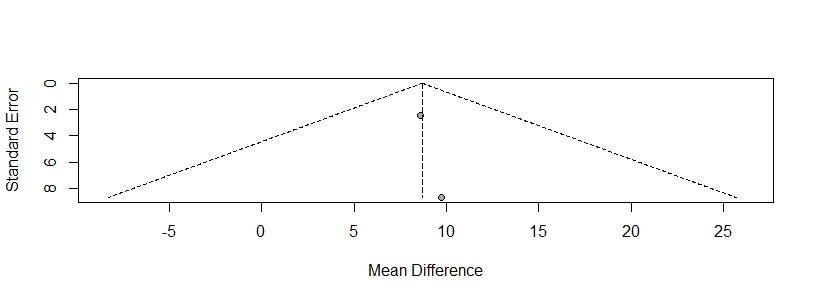

Supplement: Supplemental Information 21 — A visual assessment of potential publication bias among studies included in the meta-analysis of the maximum LRNC volumes and its correlation with MACCEs. [file peerj-14-21214-s021.jpg]

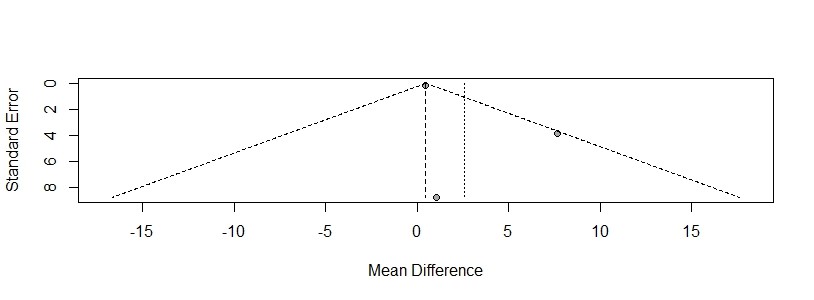

Supplement: Supplemental Information 22 — A visual assessment of potential publication bias among studies included in the meta-analysis of the proportion of LRNC volume in the vessel wall and its correlation with MACCEs. [file peerj-14-21214-s022.jpg]

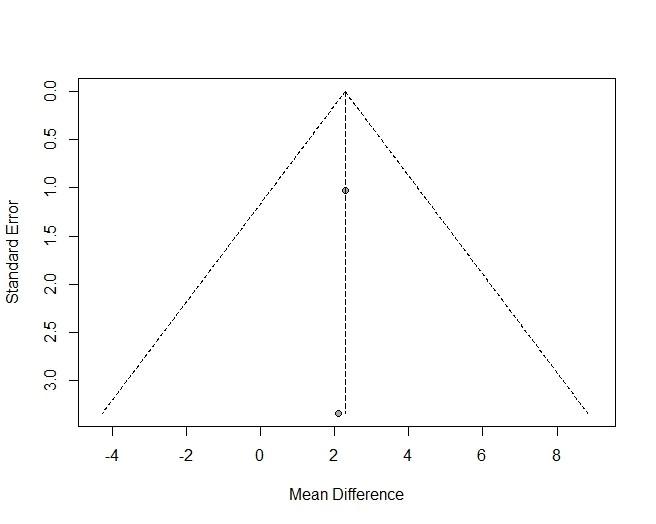

Supplement: Supplemental Information 23 — A visual assessment of potential publication bias among studies included in the meta-analysis of the proportion of LRNC volume in the vessel wall and its correlation with MACCEs. [file peerj-14-21214-s023.jpg]

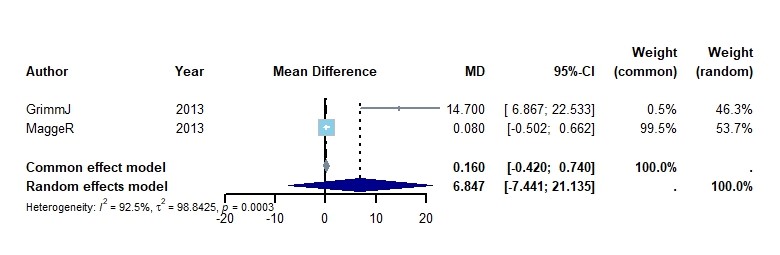

Supplement: Supplemental Information 24 — The meta-analysis results for the difference in the proportion of LRNC volume between ipsilateral and contralateral plaques in stroke patients. [file peerj-14-21214-s024.jpg]
