# Supplementary material for: Lipid-rich necrotic core of the carotid plaque and the risk of major adverse cardiovascular and cerebrovascular events: a meta-analysis and systematic review
Source: PeerJ. 2026 May 6;14:e21214. doi: 10.7717/peerj.21214 (PMC13156956; doi:10.7717/peerj.21214)
Supplement: Supplemental Information 15 — The magnetic resonance imaging sequences and plaque segmentation methodologies employed in the included studies for the identification and quantification of LRNCs. [file peerj-14-21214-s015.docx]

Supplementary Table. Summary of MRI Sequences and Plaque Segmentation Methods

| NO. | Author | Year | MRI sequences | segmentation methods | contrast use |
| --- | --- | --- | --- | --- | --- |
| 1 | TangYX | 2023 | 2D-TOF, T1W, T2W, SNAP | Visual Qualitative Assessment | No |
| 3 | BrunnerG | 2021 | 3D-TOF, 2D BB-MRI , 2D BB-MRI ce | Software-Assisted Quantitative Analysis | no |
| 4 | BosD | 2021 | 2D TOF, PDw-FSE BB, PDw-EPI, T2w-EPI, 3D T1w-GRE, 3D PC-MRA | Visual Qualitative Assessment | no |
| 5 | ToornJ | 2022 | 2D TOF, PDw-FSE BB, PDw-EPI, T2w-EPI, 3D T1w-GRE, 3D PC-MRA | Visual Qualitative Assessment | no |
| 6 | XuYL | 2016 | MERGE, SNAP, T2-VISTA | Visual Qualitative Assessment | no |
| 7 | LuMM | 2022 | 3D TOF MRA, T1W QIR, T2W DIR | Software-Assisted Quantitative Analysis | no |
| 8 | GrimmJ | 2013 | TOF-MRA, T1W, T2W, PDW, T1Wce | Visual Qualitative Assessment | yes |
| 9 | SaamT | 2016 | TOF, T1W, T2W, PDW, T1Wce | Software-Assisted Quantitative Analysis | yes |
| 10 | CheFL | 2021 | 3D-TOF, T1W QIR, T2W DIR, MPRAGE | Software-Assisted Quantitative Analysis | yes |
| 12 | HyafilF | 2016 | 3D-TOF, T1W QIR, T1W QIR CE, T2WDIR | Software-Assisted Quantitative Analysis | yes |
| 15-1 | GiannottiN | 2021 | 3D-TOF MRA, T1W QIR, PDW, T2WDIR, T1WQIR ce | Semi-Automated Quantitative Analysis | yes |
| 15-2 | GiannottiN | 2021 | 3D-TOF MRA, T1W QIR, PDW, T2WDIR, T1WQIR ce | Semi-Automated Quantitative Analysis | yes |
