# Supplementary material for: Lipid-rich necrotic core of the carotid plaque and the risk of major adverse cardiovascular and cerebrovascular events: a meta-analysis and systematic review
Source: PeerJ. 2026 May 6;14:e21214. doi: 10.7717/peerj.21214 (PMC13156956; doi:10.7717/peerj.21214)
Supplement: Supplemental Information 16 — The conventional and modified AHA histological plaque types with the presence or absence of LRNCs and their typical appearance on MRI. [file peerj-14-21214-s016.docx]

Supplementary Table. Mapping of AHA Plaque Categories to LRNC

| Conventional AHA Type (Histological) | Modified AHA Type (for MRI) | Plaque Description & Primary LRNC Relationship | LRNC Status / MRI Identification |
| --- | --- | --- | --- |
| Type I: Initial lesion | Type I-II (Combined) | Foam cells present. No necrotic core. | Absent. MRI appears as near-normal wall thickness. |
| Type II: Fatty streak |  | Multiple foam cell layers. No necrotic core. | Absent. Not distinguishable from Type I by MRI. |
| Type III: Preatheroma | Type III | Extracellular lipid pools. Precursor to LRNC, not a confluent core. | Early/Immature. Seen as focal high signal on T1/PDWI in some cases. |
| Type IV: Atheroma | Type IV-V (Combined) | Confluent extracellular lipid core (LRNC). Thin fibrous cap. | Definitively Present. Core has iso/high SI on T1WI/PDWI. MRI sensitivity=84%. |
| Type V: Fibroatheroma |  | LRNC with thick fibrous collagenous tissue. | Definitively Present. MRI cannot reliably distinguish proteoglycan vs. collagen cap (IV vs. V). |
| Type VI: Complex plaque | Type VI | Types IV or V with complication: surface defect, hemorrhage, or thrombus. LRNC often present. | Variable, often present. LRNC may be obscured by hemorrhage/thrombus. MRI detects complications. |
| Type VII: Calcified plaque | Type VII | Predominantly calcified. LRNC is typically absent or replaced. | Typically Absent. Dominant feature is calcification (low SI on all sequences). |
| Type VIII: Fibrotic plaque | Type VIII | Collagenous plaque without lipid core. | Absent. Defined by the absence of a lipid core. |
