# Supplementary material for: Lipid-rich necrotic core of the carotid plaque and the risk of major adverse cardiovascular and cerebrovascular events: a meta-analysis and systematic review
Source: PeerJ. 2026 May 6;14:e21214. doi: 10.7717/peerj.21214 (PMC13156956; doi:10.7717/peerj.21214)
Supplement: Supplemental Information 27 [file peerj-14-21214-s027.docx]

**Target Audience:** This analysis is essential for:

1. **Clinicians:** Vascular neurologists, stroke physicians, and vascular surgeons who manage patients with carotid disease for risk stratification and treatment decisions.
2. **Imaging Specialists:** Radiologists and researchers focused on plaque imaging, to standardize protocols and validate LRNC as a clinical biomarker.
3. **Guideline Developers & Researchers:** To inform future guidelines on carotid disease and inspire research into LRNC-targeted therapies.
