# Supplementary material for: Lipid-rich necrotic core of the carotid plaque and the risk of major adverse cardiovascular and cerebrovascular events: a meta-analysis and systematic review
Source: PeerJ. 2026 May 6;14:e21214. doi: 10.7717/peerj.21214 (PMC13156956; doi:10.7717/peerj.21214)
Supplement: Supplementary file 12 [file peerj-14-21214-s003.docx]

$X represents the first set of samples,$

 $Y represents the second set of samples,$ 

$Z represents the total sample,$

 $x_{1},…, x_{m}represents each data in the X sample,$

 $y_{1},…, y_{n}represents each data in the Y sample,$ 

$z_{1},…, z_{(m+n)}represents each data in the Z sample$

$\overline{z}=\frac{m\overline{x}+n\overline{y}}{m+n},$

$S_{X}=\frac{1}{m-1}\sum_{i=1}^{m}{{(x_{i}-\overline{x})}^{2}},$

$S_{Y}=\frac{1}{n-1}\sum_{i=1}^{n}{{(y_{i}-\overline{y})}^{2}},$

$S_{Z}=\frac{1}{m+n-1}[\sum_{i=1}^{m}{{(x_{i}-\overline{z})}^{2}}+\sum_{i=1}^{n}{{(y_{i}-\overline{z})}^{2}}]$ $=\frac{1}{m+n-1}[\sum_{i=1}^{m}{{(x_{i}-\overline{x}+\overline{x}-\overline{z})}^{2}}+\sum_{i=1}^{n}{{(y_{i}-\overline{y}+\overline{y}-\overline{z})}^{2}}]$ $=\frac{1}{m+n-1}[\sum_{i=1}^{m}{{(x_{i}-\overline{x})}^{2}}+2(\overline{x}-\overline{z})\sum_{i=1}^{m}{(x_{i}-\overline{x})}+m{(\overline{x}-\overline{z})}^{2}+\sum_{i=1}^{n}{{(y_{i}-\overline{y})}^{2}}+2(\overline{y}-\overline{z})\sum_{i=1}^{n}{(y_{i}-\overline{y})}+n{(\overline{y}-\overline{z})}^{2}]$ $=\frac{1}{m+n-1}[(m-1)S_{X}+m{(\overline{x}-\overline{z})}^{2}+(n-1)S_{Y}+n{(\overline{y}-\overline{z})}^{2}]$ $=\frac{1}{m+n-1}[(m-1)S_{X}+(n-1)S_{Y}+m{(\frac{n(\overline{x}-\overline{y})}{m+n})}^{2}+n{(\frac{m(\overline{y}-\overline{x})}{m+n})}^{2}]$ $=\frac{1}{m+n-1}[(m-1)S_{X}+(n-1)S_{Y}+\frac{mn}{m+n}{(\overline{x}-\overline{y})}^{2}].$
